# A plausible mechanism for auxin patterning along the developing root

**DOI:** 10.1186/1752-0509-4-98

**Published:** 2010-07-21

**Authors:** Victoria V Mironova, Nadezda A Omelyanchuk, Guy Yosiphon, Stanislav I Fadeev, Nikolai A Kolchanov, Eric Mjolsness, Vitaly A Likhoshvai

**Affiliations:** 1 Institute of Cytology and Genetics, SB RAS, Lavrentyeva 10, Novosibirsk, Russia; 2 Novosibirsk State University, Pirogova 2, Novosibirsk, Russia; 3 Institute of Mathematics, SB RAS, Koptjuga 4, Novosibirsk, Russia; 4 Department of Computer Science, University of California, Irvine, USA; 5 Institute for Genomics and Bioinformatics, University of California, Irvine, USA

## Abstract

**Background:**

In plant roots, auxin is critical for patterning and morphogenesis. It regulates cell elongation and division, the development and maintenance of root apical meristems, and other processes. In *Arabidopsis*, auxin distribution along the central root axis has several maxima: in the root tip, in the basal meristem and at the shoot/root junction. The distal maximum in the root tip maintains the stem cell niche. Proximal maxima may trigger lateral or adventitious root initiation.

**Results:**

We propose a *reflected flow *mechanism for the formation of the auxin maximum in the root apical meristem. The mechanism is based on auxin's known activation and inhibition of expressed PIN family auxin carriers at low and high auxin levels, respectively. Simulations showed that these regulatory interactions are sufficient for self-organization of the auxin distribution pattern along the central root axis under varying conditions. The mathematical model was extended with rules for discontinuous cell dynamics so that cell divisions were also governed by auxin, and by another morphogen *Division Factor *which combines the actions of cytokinin and ethylene on cell division in the root. The positional information specified by the gradients of these two morphogens is able to explain root patterning along the central root axis.

**Conclusion:**

We present here a plausible mechanism for auxin patterning along the developing root, that may provide for self-organization of the distal auxin maximum when the *reverse fountain *has not yet been formed or has been disrupted. In addition, the proximal maxima are formed under the *reflected flow *mechanism in response to periods of increasing auxin flow from the growing shoot. These events may predetermine lateral root initiation in a rhyzotactic pattern. Another outcome of the *reflected flow *mechanism - the predominance of lateral or adventitious roots in different plant species - may be based on the different efficiencies with which auxin inhibits its own transport in different species, thereby distinguishing two main types of plant root architecture: taproot vs. fibrous.

## Background

Plant architecture is formed by the activities of meristems, which comprise stem cells and their derivatives, giving rise to various cell types. The root apical meristem (RAM) is formed at the earliest stages of embryogenesis and is localized to the root apex after germination [1]. Depending on the dominance of the primary root, two main types of the plant root architecture are classified as taproot and fibrous. Mechanisms determining root architecture and mechanisms for stem cell niche maintenance in RAM are often considered to be separate. However, accumulating evidence concerning the primary role of auxin transport in both processes (reviewed in [2]) suggests that they can be united into a single system, the structural features and dynamics of which can be described by one mathematical model. Three types of auxin concentration maxima in the root have been experimentally detected: (1) in the RAM, namely, in the root cap initials, with a decreased level in the quiescent center (QC) and root cap [3] (here auxin regulates stem cell niche maintenance); (2) in the protoxylem cells of the basal meristem (upper boundary of the meristematic zone) and the pericycle of the root differentiation zone - at the sites of lateral root predetermination and initiation, respectively [4]; and (3) at the shoot-to-root junction which includes sites of adventitious root initiation [5].

Auxin concentration maxima in plant tissues are mainly formed due to active auxin transport between cells [6-9]. Polar-localized auxin carrier proteins form auxin fluxes in the tissue (reviewed in [10]). The auxin synthesized in the shoot is acropetally transported through the vascular system towards the root tip, whereas the oppositely directed (basipetal) flow goes through the epidermis (reviewed in [11]). It has been demonstrated that PIN family efflux carriers are the main contributors to the formation of the auxin distribution pattern in the root [7,12]. In particular, the PIN1, PIN3, PIN4, and PIN7 proteins provide for a continuous auxin flow along the apical-basal root axis via the vascular system to the QC cells. PIN3 and PIN7 are also involved in the lateral redistribution of auxin in the root cap. PIN2 proteins mediate basipetal auxin transport from the root tip via the epidermis as well as acropetal auxin transport in cortex.

Understanding of the role of active transport in the formation of auxin concentration gradients is a topical problem in developmental biology. Computer modeling approaches are also used for solving this problem. In particular, Jonsson et al. (2006), Smith et al. (2006), and de Reuelle et al. (2006) have studied models for phyllotaxis mechanisms in the shoot [13-15]; Stoma et al. (2008) studied a model for mechanisms of auxin transport regulation in shoot and root meristem development [16]. Grieneisen et al. (2007) developed a model for auxin gradient formation in the root tip [17]. The model is based on the concept of a "reverse fountain", proposed by Swarup and Bennet (2003) [11] and experimentally established by Blilou et al. (2005) [7]. According to this concept, the acropetal and basipetal auxin flows are coordinated to generate and maintain an auxin distribution in the root tip (reviewed in [18]). In Grieneisen et al. (2007) the concept was formalized as a two-dimensional computer model of auxin transport in the root [17]. In this model, the root tissue is represented as a structured cell layout with different localization of the PIN family proteins in four cell types. A stable location of the auxin maximum *in silico *is provided for by a reflux of auxin from the basipetal flow back to the acropetal flow all along the meristem, which transports auxin in a loop.

The *reverse fountain *mechanism is based on a pre-assigned positioning and levels of PINs in RAM. Such a mechanism, whereby tissue patterning predetermines the morphogene distribution, could be defined as a "structural mechanism". Structural mechanisms describe well the processes of auxin distribution in a mature root - in pre-established RAM structure [17] or in the curved root regions where lateral roots form [19]. Despite these features of structural mechanisms, they are not applicable to formation of an auxin gradient in cases where the root structure has not yet been formed or has been disrupted: in the basal part of the embryo, in the undeveloped meristems of the main and lateral roots, and during RAM regeneration. Indeed, *the reverse fountain *structural mechanism requires the presence of three flux types-acropetal, lateral, and basipetal. The PIN2 proteins, which are responsible for the basipetal flow, are not expressed at the early stages of development of the primary [20] and lateral [8] roots. The *reverse fountain *mechanism also cannot function in the root immediately after damage of RAM structure by a laser ablation. After QC laser ablation the columella is destroyed [21], so that the lateral redistribution mechanism is impaired and, as a result, the basipetal auxin flow doesn't receive its auxin supply. During subsequent RAM regeneration, the auxin maximum appears first in the root vascular cells and only then the QC and root tip structure are regenerated [3,21]. Formation of auxin maxima in the basal meristem, which predetermine lateral root initiation in the pericycle of the root differentiation zone [4], also occurs in a cell environment lacking any structural elements that could implement the structural mechanisms. Thus, there must exist some mechanisms that form auxin concentration maxima and act before the establishment the root structure, and subsequently act in parallel with the *reverse fountain *mechanism.

In a number of works, it has been shown that the formation of an auxin gradient precedes tissue patterning (reviewed in [22]). Thus, the self-organization mechanisms that determine the auxin distribution pattern are important for the morphogenetic mechanisms to work. In this paper, we propose and substantiate an alternative mechanism for auxin distribution pattern formation in the developing root. For definiteness, we called it the "*reflected flow*" mechanism. The name "reflected flow" suggests the dynamics by which the auxin maximum gets positioned a few cells away from the root end, as described in the Results section below. The mechanism explains self-organization of the auxin distribution pattern in an array of functionally identical cells acquiring cell type specialization due to auxin regulation of the level of PIN proteins in these cells, although the orientation of the PIN is assumed already to be established. A similar mechanism for PIN allocation was used in the shoot apical dominance model presented by Prusinkiewicz et al. (2009) [23]. It has been recently shown that auxin controls expression of its carriers, both influx and efflux. Positive as well as negative regulation was shown in a number of experiments. Auxin activates transcription of PIN family genes via the Aux/IAA-ARF signaling pathway [24]. Polar localization of PIN proteins on the cell membrane may be also regulated by auxin [25]. The negative feedback from auxin to its rate of transport is provided by an increased degradation of the PIN proteins, which is observed at high auxin concentrations [24].

Here, we present a mathematical model that implements the *reflected flow *mechanism for formation of the auxin distribution pattern along the root. This model describes (1) auxin flow from the shoot, which is a sole source of auxin; (2) irreversible loss (degradation) of auxin through its utilization or migration from the modeled region; (3) auxin diffusion, providing for an isotropic distribution in the root; (4) active auxin transport in the direction from the shoot to the root tip (acropetal flow), which is regulated by the PIN1 protein; (5) synthesis and degradation of PIN1 protein within cells, depending on auxin concentration in each cell; (6) growth and division of root cells. The rate of cell division in the model is regulated by auxin and a hypothetical *Division Factor *so that the distribution of cell divisions along the *in silico *root qualitatively matches the profile of mitotic activity observed experimentally.

We will demonstrate that the *reflected flow *mechanism accounts for (1) the formation of the auxin concentration maximum in the root tip from the initially unspecialized tissue and (2) the maintenance of the auxin maximum during early root development. The positional information specified by the gradients of morphogens auxin and *Division Factor *makes it possible to differentiate cells of different types and to explain the RAM patterning. We will reproduce *in silico *the experimental data on the changes in auxin distribution pattern after (1) root tip cut or QC laser ablation, (2) root exposure to inhibitors of active auxin transport, and (3) root treatment with exogenous auxin. Our simulations also will show that an increase in the auxin flow from the shoot to the root results in the formation of additional auxin concentration maxima in the regions corresponding to the basal meristem and the shoot-to-root junction, where the lateral and adventitious roots, respectively, are initiated. Based on an analysis of the model with various sets of parameters, we propose the following hypothesis on the key role of self-inhibition of auxin transport in the development of different root architectures. The taproot system (dominance of the primary root, rare adventitious roots and lateral roots located at a certain distance from one another) develops *in silico *at a high threshold for the auxin-induced degradation of PIN proteins. By contrast, the fibrous root system (termination of the primary root, the massive production of adventitious and lateral roots) develops in the case of a low threshold, i.e., if the degradation rate for PIN proteins grows rapidly with increasing intracellular auxin concentration.

## Methods

### Simulation of Auxin Distribution in Root

#### Biological assumptions

The acropetal transport is of dominant importance in supplying auxin to the root in the early stages of seedling development, until the root gains competence to synthesize its own auxin about 5 days after germination [26]. Therefore, in the model we take into account the auxin flow from the shoot to root as the sole source of auxin in early root development.

Of all the auxin carriers, we currently only consider PIN1. PIN1 is expressed in the root vasculature and weak expression is sometimes observed in the QC [24]. The expression domain and polar localization of this efflux carrier correspond to the zone of active acropetal flow. As *PIN2 *is not expressed at early stages of root development [8,20], we do not consider basipetal flow in the 2D model.

It has been shown that at low concentrations, auxin activates transcription of *PIN1*, whereas at high auxin concentrations, an increased degradation of the corresponding protein is observed [24]. The mechanisms underlying the regulation of *PIN1 *expression are complex, and not all the details are known. So we modelled the effect of auxin on *PIN1 *expression by approximating functional forms, in what follows.

#### Mathematical description

##### I. Elementary processes in the model of auxin distribution

Auxin redistribution along the central root axis is described based on the interactions of a limited set of dynamic intracellular processes, namely:

Auxin flow from the shoot to the root

The rate of auxin flux into the cell is described by the following rate equation:(1)

where *α *is the intensity of auxin flux from the shoot, and *a *is auxin concentration that is normalized to *V_a _*and measured in concentration units (*cu*);

Auxin degradation

The conjugation, oxidation, and lateral distribution of auxin are summarized in the generalized degradation process. The rate of auxin degradation in the cell is described as(2)

where *K_d _*is the degradation rate constant.

Auxin diffusion

The rate of auxin diffusion from one cell to another is described as(3)

where *D *is the diffusion rate constant.

Active auxin transport

The rate of active transport via the PIN1 proteins (*PIN*, concentration of PIN1 proteins, that measured in *cu*) is described by the following mass action equation:(4)

where *K_0 _*is the constant of active transport rate.

Expression regulation of the PIN1 protein

**Figure 1:**
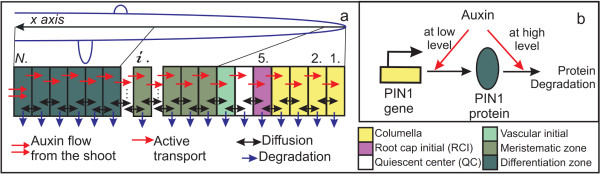
**Representation in the model the processes influencing the auxin distribution along the central root axis**. a. Acropetal flow is considered in the model along the cell array on the central root axis (*x *axis). Arrows denote the processes that provide for auxin movements considered in the model. b. The summarized mechanism providing for regulation of PIN1 expression comprises the regulation of PIN1 protein synthesis and degradation depending on the auxin concentration.

PIN1 concentration dynamics in individual cell was defined by auxin-dependent rates of PIN1 synthesis and degradation (Figure 1B). The rate of PIN1 protein syntheses was approximated by the following Hill function:(5)

This function is zero at zero auxin concentration in a cell and monotonically increases to  as a function of auxin concentration.

The rate of PIN1 protein degradation is modelled as the rational polynomial, monotonically increasing as a function of its arguments *a *and *PIN*:(6)

In Eqs. (5) and (6) the parameter *k_1 _*is the synthesis rate constant; *q_1 _*is the threshold of auxin-dependent activation of PIN1 synthesis; *q_2 _*is the threshold for saturation of auxin-dependent PIN1 synthesis; *k_2 _*is the rate constant for basal level degradation of PIN1 protein; *q_3 _*is the threshold of auxin-dependent PIN1 degradation; and *h*_*1 *_and *h_2 _*are coefficients, which determine the response rate of these processes to the changes in intracellular auxin concentration.

PIN1 protein localization on cell membranes

The biological and mathematical description of PIN1 polarization mechanisms is an important scientific challenge, but it is beyond the scope of this work. We consider an array of functionally identical cells, where PIN1 proteins are polarly localized at one cell side, mediating active auxin transport only in the acropetal direction.

##### II. Description of the 1*D minimal model *for auxin distribution along the root

The one-dimensional (1*D*) model describes auxin distribution in a linear array of cells located along the central root axis (Figure 1). At time *t*, we number the cells within the modeled zone in the following manner: number 1 is ascribed to the last cell in the line of the model, that corresponds to the cell of the outer layer of the columella root cap. The remaining cells are numbered from 2 to *N *in the direction from root end to its base. Thus, the cell of the model corresponding to the cell at shoot-to-root junction has number *N*. Processes (2)-(6) are specified identically for each cell (except cells 1 and *N *which must have boundary conditions imposed), in such a way that every cell within the linear array actively transports auxin towards the first cell, thus forming the auxin acropetal flow.

Auxin from the shoot first enters the *N*th cell and then spreads through the linear array of cells by diffusion and active transport. Therefore, process (1) (auxin inflow from the shoot) is specified only for the *N*th cell. On the other hand, irreversible loses of auxin are defined for all cells in the array according to Eq. (2).

Diffusion is regarded as an isotropic process of auxin movement from the current cell to both preceding and next cells. Eq. (3) is used to describe the diffusion along the central root axis. For cell 1, passive diffusion is necessarily defined only to cell 2 due to the physical boundary conditions - there being no adjacent cell in the other direction. For cell *N *the boundary condition is that the net effect of active transport and passive diffusion is defined as occurring in only one direction: from the unmodeled shoot toward the root tip, modeled as process (1). Thus, processes (3) and (4) are active only acropetally in cell N. Diffusion to cells located laterally but beyond the modeled zone is roughly taken into account in the auxin degradation process (2).

We consider that it is possible for PIN1 protein to be synthesized and degraded in each cell of the array, in an auxin concentration-dependent manner. (Figure 1B). These processes of regulated PIN1 synthesis and decay are described by Eqs. (5) and (6). PIN1-mediated active transport is specified as an anisotropic process of transport from the *i*th cell to cell *i - *1. This process of active transport is described by Eq. (4). Active transport is not considered in the first cell due to its physical boundary condition.

Summarizing all these assumptions, we get the following system (hereinafter, the 1*D minimal model*):(7)

In model (7), *a_i _*and *PIN_i _*denote the concentrations of auxin and PIN1 in the *i*th cell.

##### *II*. Description of the 2*D minimal model *for auxin distribution in the root tip

**Figure 2:**
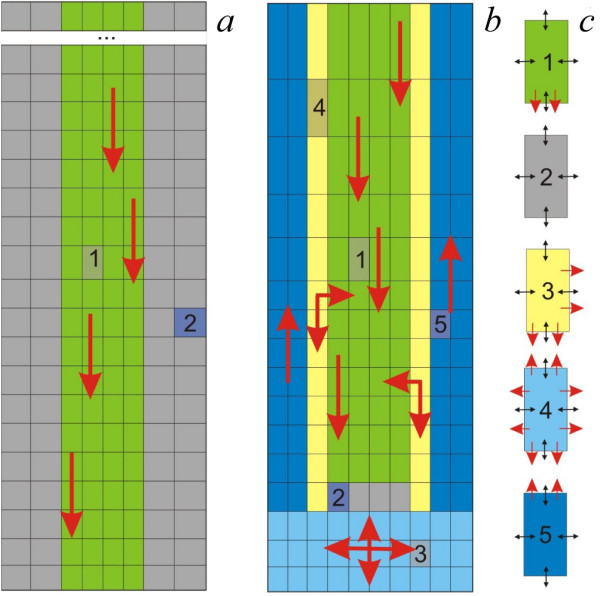
**The cell layouts in the 2D models of auxin distribution**. a. The cell layout in the *2 D minimal model *represented by two layer types - provascular (green) and epidermal (gray). b. The cell layout of the model described in [17]. c. The whole set of cell types considered in the models (a, 1 and 2) and (b, 1-5). Red unidirectional arrows in (c.) mark the directions of active auxin transport for each kind of cell. Black bidirectional arrows mark the auxin exchange by diffusion. Auxin flows in a.-b. are delineated by thick red arrows.

The two-dimensional (2*D*) model describes auxin distribution in a cell layout representing a longitudinal cut of root at early developmental stages. The rectangular cell layout consists of *M *layers (*j *= 1, ..., *M*) of *N *cells each (*i *= 1... *N*). In the present paper we show data on 2D model calculations with *M *= 8 and *N *= 50 (Figure 2A, C). The auxin and PIN1 concentrations in the cell located in the *i*th cell of the *j*th layer are described by variables *a*_*j, i *_and *PIN*_*j, i*_, respectively. Every inner layer of the cell layout for varying index *i *and fixed *j*,) *j *∈ {3, ..., *M *-2})corresponds to a linear cell array located along the central root axis. We refer to these layers as "provascular" layers. The auxin and PIN1 dynamics in the provascular layers are described by the ordinary system equations (7), identical to that describing the 1*D minimal model *(where the variables *a*_*i *_and *PIN*_*i *_are changed to *a*_*j, i *_and *PIN*_*j, i *_for the *j*th layer). Additionally to the lengthwise processes described by (7), for the provascular layers we included the transverse processes of auxin exchange by diffusion between the adjacent cells in the neighboring layers. We model the transverse diffusion by equation (3), which is the same as for the longitudinal diffusion. Taking into account the double indexing of dynamical variables, the transverse diffusion in the inner layers of the 2 D model is described by the rate function:(8)

The outer "epidermal" layers, *j *∈ {1, 2, *M *-1, *M*} of the 2D model correspond to epidermal layers of root at early stages of development. The characteristic of the layers is their inability to synthesize (5) or degrade (6) the PIN1 protein in their cells. As basipetal auxin flow is not engaged in early root development [8,20], we don't include active auxin transport (4) in these layers. Auxin moves along as well as between these layers only by diffusion, process (3). We also do not consider the auxin flow from the shoot (1) to the epidermal layers, so that the sole source of the auxin to the epidermal layers is the transverse diffusion from the adjacent provascular layers *j *∈ {3, *M *-2}. Auxin degradation (4) is defined for all cells in epidermal layers. The transverse diffusion (3) in the outermost epidermal layers (*j *∈ {1, *M*}) is described taking into account their boundary position. The system of ordinary equation that describes auxin dynamics in *j *∈ {1, *M*} outer epidermal layers is the following:(9)

where *δ*_1 _= +1, *δ_M _*= -1. The full system of ordinary equations for the 2*D minimal model *that describes the processes (7), (8), and (9) in the *M*x*N *cell layout is presented in [Additional file 1: Text S1].

Comparing the cell layout described in Grieneisen et al., (2007) [17], the 2*D minimal model *doesn't contain any structural elements that could provide for the function of the *reverse fountain *mechanism (Figure 2). However, unlike [17], this model does provide for the dynamics of the quantitative degree of polarized PIN through the level of PIN1 expression in each provascular cell.

### Modeling of Root Cell Growth and Division

#### Biological Background

**Figure 3:**
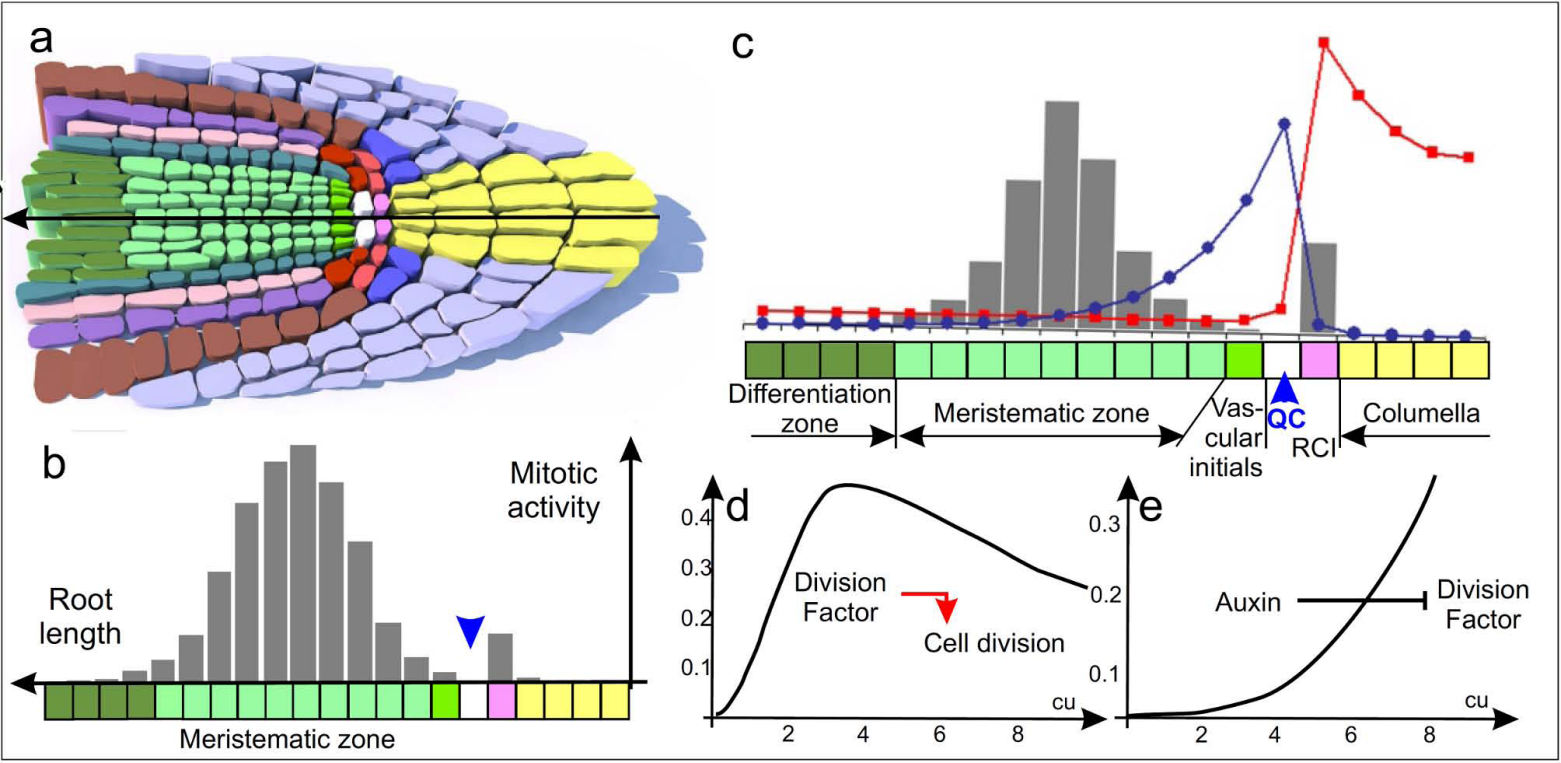
**Mitotic activity in the root and its simulation**. a. The scheme of root tip structure in Arabidopsis. The cells of different types marked by different colors (for details, see figure 1). b. Qualitative profile of mitotic activity in cells along the central root axis. Two maxima of mitotic activity are distinguished along the central root axis according to Dolan et al. (1993) [1] and Beemster and Baskin (2000) [27]. c. The model solution: auxin (red squares) and substance Y (blue circles) distributions regulate the rates of cell divisions in the root (gray columns). The dynamical characteristics (cell coordinates on the axis, auxin concentration, and division rates) in the model solutions reproduce root patterning, where the cells of different types are specifying around the distal auxin maximum. d. The plot of auxin-regulated Y degradation rate used in the model (Eq. (8)). (E) The plot of Y-regulated rates of cell division (first coefficient in Eq. (10)). QC is the quiescent center and RCI is the root cap initial.

The profile of cell mitotic activity along the meristematic zone of the root is bell-shaped with the maximum located at a distance of 10-16 cells from the QC [27]. Taking into account also the dividing root cap initials [1], the profile of mitotic activity in the whole root acquires two maxima of the division rate along the central root axis (Figure 3A, B).

Cell divisions in root are governed by different hormones. Depending on the concentration, auxin acts in different fashions on the cell division rate: low and high auxin concentrations have a negative effect, whereas its intermediate concentrations has a positive effect [28], thereby making the curve of dose dependence bell-shaped. Ethylene can activate cell divisions in RAM [29], whereas cytokinin mainly inhibits rates of cell divisions [30]. In the model, we introduced a *Division Factor *that combines functions of these hormones to regulation of cell division rates.

#### Mathematical description

##### I. Dynamical grammar for more realistic cell dynamics simulation

To find out whether a more realistically cellularized model that includes cell growth and cell division dynamics might disrupt or destroy the pattern formation process, the 1*D minimal model *(7) was expanded using the formalism of Dynamical Grammars (DG; [Additional file 1: Text S2], [31-33]). We specify the *x *axis as the central root axis directed towards the root base with the origin at the point corresponding to the outer wall of cell 1. For each cell, we consider the size *r*, which it occupies along the *x *axis, and the coordinate of its center on the *x *axis. Cell division and death (sloughing of the root cap cells) are described as discrete events in the formalism of DG stochastic rules [31]. The cell growth and the corresponding change in the positions of cells along the *x *axis are described by differential equations. The processes of auxin distribution in the linear cell array (7) were rewritten according to the DG syntax (for details, see [Additional file 1: Text S2]). The model of auxin distribution along the central axis of the *in silico *growing root in DG formalism is hereinafter referred as the *1D extended model*.

##### II. Description of cell cycle in the model

The cell cycle in the model is described in the formalism of DG stochastic rules as a sequence of two phases: growth phase and idle phase. The moments of growth and idle phase completion are random variables with probability density functions depending on the functions *f_GP _*and *f_IP_*, respectively (for the details see [Additional file 1: Text S2]). Cell dynamics in the *1D extended model *is described by the following elementary processes:

Cell growth

Increase in cell length *r *on the *x *axis in time *t *takes place only in the growth phase and is described as rate function:(10)

During the idle phase the cell does not grow, so *r *= *r*_*0*_+*K*_*growth*_*τ *= *const*, where *τ *is the realized growth phase duration.

Duration of the growth phase

The growth phase in the cell commences immediately at time *t*, when the cell appears in the modeling zone through division of the mother cell into two daughter cells. The function of growth phase completion *f_GP_(r) *is dependent on the current cell size *r*:(11)

where *r_min _*is the minimal cell size that allows the cell to divide. The *f_GP _(r) *function defines the probability of growth phase completion, which increases with time. At the moment of growth phase completion, the cell enters idle phase.

Duration of the idle phase

The idle phase duration defines the rate of cell division. In the model the function of the idle phase completion *f_IP_*(*DivF*) depends on concentration of the *Division Factor *(*DivF *) in a cell:(12)

The values of parameters in Eq. (12) are selected so that the idle phase is be longer in case of either deficiency or excess in the *Division Factor *and shorter at medium values of its concentration (Figure 3D), making the curve of dose dependence bell-shaped. (This is possible since there are two exponent parameters instead of one, as there would be in a conventional Hill function. Generalized Hill functions that can be specialized to this form are discussed in [34]. The end of idle phase means that the cell has divided into two daughter cells. At this moment, the cell returns to growth phase. At the moment the daughter cell appears, it has size *r_0 _*corresponding to half that of the mother cell before division. After division the cells in the *1D extended model *are effectively renumbered according to their changed order on the *x *axis.

##### II. Formation of the gradient of *Division Factor *in the model

To simulate a realistic cell dynamics along the central root axis (Figure 3A, B) using the *Division Factor *as a regulator and repressor of cell division, we defined a set of requirements for the *Division Factor *distribution. First, *Division Factor *must have a maximum in the cell next to the maximum of auxin concentration (QC) where it greatly inhibits cell division. Second, *Division Factor *must be negligibly low in cells located proximally at a certain distance from QC (differentiation zone). Third, the *Division Factor *must have similar moderate concentration levels in the cell with maximal auxin concentration (the root cap initial) and in the cells located in certain interval proximal to the QC (the meristematic zone). To satisfy all these requirements, the self-organization of the *Division Factor *distribution is provided by the following set of intracellular processes.

The synthesis of Division Factor

Synthesis of the *Division Factor *in the *i*th cell with the rate *V*_*DivF*_(*a*_*i*_, *a*_*i*+1_) depends on the difference in auxin concentration in the cell compared to the (*i*+1)th cell:(13)

where constant *β *is the maximal level of *Division Factor *synthesis and parameter *T *= 0.1. The sigmoidal function *V_s, DivF_*(*a*_*i*_, *a*_*i*+1_) grows monotonically in the interval [0;1] as the *a*_*i*+1 _-*a*_*i *_gradient increases. Due to the boundary position of the cell *i *= *N*, the auxin gradient is not defined in this cell, so there is no synthesis of *Division Factor *in the cell.

Division Factor degradation

The rate of *Division Factor *degradation *V_d, DivF_*(*a*) in the cell depends on the auxin concentration within it (Figure 3E) and is described by a generalized Hill function as:(14)

where  is the rate coefficient for auxin-dependent degradation of *Division Factor*;  is the threshold of auxin-dependent activation of *Division Factor *synthesis;  is the threshold of auxin-dependent saturation of *Division Factor *synthesis; *h_3 _*and *h_4 _*are Hill coefficients which determine the response rate of these processes to the changes in intracellular auxin concentration.

Division Factor diffusion

The rate of *Division Factor *diffusion from one cell to another is described as(15)

Where *D_DivF _*is the diffusion rate constant.

The dynamics of *Division Factor *concentrations in the cell array is described in ordinary differential equations as:(16)

In Eq. (16), *DivF_i _*denotes the concentration of *Division Factor *in the *i*th cell. The concentration gradient of *Division Factor *generated by (16) defines the positional information that governs the profile of cell divisions along the root.

### Practical aspects of modeling

#### Numerical solution

The nonlinear system of equations of 1*D *and 2*D minimal models *was integrated using the MGSModeller software package [35]. The *minimal models *was solved using Gear's method [36]. The multiplicity and stability of stationary solutions for the model were studied using the parameter continuation method in STEP+ software [37]. The 1*D extended model *[Additional file 1: Text S2] executes in Plenum [Additional file 2], an implementation of DG within *Mathematica*, a computer system for symbolic mathematics [31-33]. The 1*D extended model *in *Mathematica *was solved using Runge-Kutta 4th order method, the results of the 1*D minimal *and 1*D extended models *with the same sets of parameters and cell numbers were similar (data not shown).

#### Parameter estimations

The parameters were estimated using published experimental data on the mechanisms involved in the regulation of *PIN *expression and auxin dynamics in the cell [Additional file 1: Text S3]. Several parameters were determined by the coordinatewise descent method [Additional file 1: Text S3].

#### Verification of model calculation results

The proposed model is based on published data. The experimental data on the auxin distribution in the root are represented by images of roots from transgenic *DR5*::*GUS *plants, for example in [3,7,8]. Expression of DR5 transcriptional fusions has been shown to be proportionally responsive to a range of auxin concentrations [3]. The plots of staining density distribution for the products of reporter gene *DR5*::*GUS *along the central root axis were reconstructed by processing the images using the ImageJ software package [38] see for details [Additional files 1: Text S3; 3].

## Results

### Mechanisms of Auxin Acropetal Transport Regulation are Sufficient for Self-organization of Auxin Distribution Pattern in Roots

**Figure 4:**
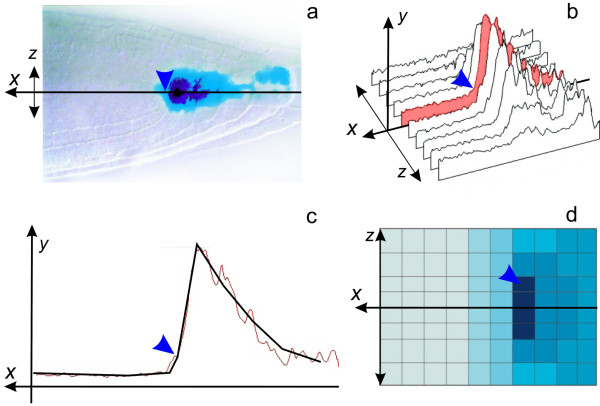
**The auxin distribution pattern reproduced by the model**. a. Expression of DR5::GUS detects the auxin pattern in the root tip (adapted from Sabatini et al., 1999; [3]). b. The surface plot of DR5 activity in the root tip scanned from figure 2a by ImageJ program. The *x *axis corresponds to the central axis of the root; the root width extends along the *y *axis; and the *z *axis shows DR5 activity. c. The model solution (black line) agrees well with semi-quantitative data of auxin distribution along the central root axis, obtained from the surface plot at figure 2b (red line). d. The auxin distribution pattern in the stationary solution of the 2*D minimal model*. Blue arrowhead denotes the QC position.

A characteristic feature of auxin distribution patterns in the root is the presence of concentration maximum in the root cap initial cells (Figure 4A, B; [3]). We have hypothesized that auxin regulation of its own transport with positive and negative feedbacks (Figure 1B) is a sufficient condition for formation of this characteristic auxin distribution pattern in a functionally uniform cell array. To verify this hypothesis, we constructed 1*D *and 2*D minimal *mathematical models that take into account auxin and PIN1 concentrations dynamics (see the Methods section). The 1*D minimal model *(7) describes the auxin distribution (from a source in the shoot) over a linear array of non-dividing cells located along the central root axis (Figure 1A). The same processes take place in each cell of the model except for the cells at both ends of the root due to necessary boundary conditions. For the 1*D minimal model *with number of cell *N *= 50, we estimated several sets of parameter values that gave steady-state solutions with auxin distribution matching the experimentally observed pattern reported by Sabatini et al. (1999) ([3]; Figure 4B-C; [Additional files 1: Text S3; 3; 4: I]). Two of them, the "basic" [Additional file 4: II] and the "robust" [Additional file 4: III] are used in the present work for the model analysis.

The mechanism of auxin distribution self-organization found in the resulting stationary solutions is the following. In cells with low auxin concentration, the positive regulation of PIN1 expression by auxin provides for self-enhancement of the acropetal auxin flow. This results in a rapid auxin accumulation at the root end (cell number 1). This is followed by the increase of auxin concentration in the neighboring cell 2 whereto high amount of auxin moves by diffusion from the cell 1 (reflected flow of diffusion). As soon as the auxin concentration in cell 2 exceeds the threshold for auxin-dependent PIN1 degradation (*q_3_*), auxin starts to inhibit PIN1 expression. This leads to a decrease in the active auxin transport towards cell 1 and a shift in auxin maximum from cell 1 to cell 2. Subsequently the same processes occur in cell 3, cell 4, etc. leading to a corresponding shift of the auxin maximum away from the end of the root. This shift continues until the acropetal and reflected auxin flows becomes balanced. For definiteness, we named this mechanism of auxin maximum self-organization the *reflected flow *mechanism.

The *reflected flow *mechanism was also examined in the 2*D minimal model*. The 2*D minimal model *simulates auxin distribution in the root at early stages of development (no auxin synthesis and basipetal auxin flow; for the details see the Methods section). The cell layout of the 2D model is a rectangular array that consists of four "provascular" inner layers and two "epidermal" outer layers on each side of the provascular layers (Figure 2A). The *2 D minimal model *simulation with the basic set of parameters yields a stationary solution with (1) an auxin maximum in the provascular layers at a distance from the root end and (2) auxin gradients in the epidermal layers from the root end towards the root base (Figure 4D). Comparing this situation to the *reverse fountain *mechanism for auxin maximum formation in the root tip (Figure 2B), the *reflected flow *mechanism doesn't require the following anatomical elements and specific PIN localization within them to be pre-assigned: (1) root cap cells where PIN proteins redistributes auxin in all directions; (2) basipetal auxin flow in the epidermal layers; (3) lateral auxin transport at the edge between the vascular cylinder and epidermal layers, that accounts for the auxin reflux from the basipetal flow back to acropetal flow in the *reverse fountain *mechanism. In the *reflected flow *mechanism, the auxin maximum in the root tip arises from the dynamics of the level of PIN1 in an auxin concentration-dependent manner (Figure 1B). Only one anatomical element has to be preexisting for the operation of the *reflected flow *mechanism - provascular tissue with polarized PIN protein localization, which provides for unidirectional auxin flow (Figure 2A). This circumstance naturally occurs in the root tip during early stages of RAM development and also during RAM regeneration. Consequently, the results obtained here favor the *reflected flow *mechanism of auxin distribution pattern self-organization over the *reverse fountain *mechanism in cases where the RAM structure has not yet developed or has been disrupted.

### Maintenance of auxin maximum in a growing root

To investigate whether cell divisions might disrupt or destroy the auxin distribution pattern that arises under the *reflected flow *mechanism, we created the 1*D extended model *of auxin distribution with cell growth and dynamics (See the Methods section). For the 1*D extended model*, we estimated the values of additional parameters that were introduced when expanding 1*D minimal model*. In this way, we obtained a parameter set that guaranteed that the 1*D extended model *also provides for formation and maintenance of the auxin distribution pattern in the *in silico *growing root [Additional files 1: Text S3, 5]. Note also that the additional condition for formation and maintenance of auxin pattern during the root growth is coordination of growth with the increase the rate of auxin flow from the shoot to the root (*V*_*α*_) The root growth in the 1 D *extended model *with the basic set of parameters was simulated starting from three cells, and specifying the initial auxin maximum in the second cell, which corresponds to the auxin distribution pattern in the embryonic RAM of *A. thaliana *[12]. When simulating the root growth from the three initial cells, in short roots the auxin concentration maximum is localized to the second cell. With increase in root length, the distance from first cell to the maximum increases to four cells and then remains constant [Additional file 6]; these changes in the auxin distribution qualitatively match experimental observations [12]. In a root that exceeds about 150 cells in length the maximum shifts towards the root end and then disappears, even if we greatly increase *V*_*α*_. This effect can be avoided by adding to the model processes of self-regulated auxin synthesis (data not shown). Consistent with this *in silico *observation, *in vivo *at the early stages of seedling development the shoot is the main source of auxin to root, but as the root develops the auxin synthesized in the root becomes of dominant importance [26]. Thus, study of the 1*D extended model *has demonstrated that cell growth and division in the root do not interfere with the formation and maintenance of the distal auxin maximum.

### Root Apical Meristem Patterning Along the Central Root Axis

An additional morphogen was introduced to the 1*D extended model *to simulate more realistic cell-level dynamics along the central root axis (Figure 3A, B), including cell growth and cell division. The *Division Factor *combines functions of ethylene and cytokinin hormones to regulation of cell division, its synthesis and degradation rates depend on auxin (see Methods section). When calculating the 1*D extended model*, the concentration maxima of the morphogens auxin and *Division Factor *are localized to the neighboring cells at an approximately constant distance from the root tip (Figure 3C). Characteristic of *Division Factor *distribution pattern is a smoother decrease in its concentration towards the root base as compared with the opposite direction to the root tip. Eventually, a pattern of morphogens along the root is established in the model that can be interpreted in terms of the theory of positional information. The local concentrations of morphogens and their gradients generated *in silico *make it possible to calculate the rates of root cell division and compare the resulting dynamic characteristics (cell coordinates on the axis, auxin concentration, and division rates) with the characteristics of cell types located *in vivo *along the central root axis.

Consequently, we obtain *in silico *the following structure (Figure 3C):

*(i) *Three to four nondividing cells with a high auxin concentration are located in the root end. Their characteristics match the characteristics *in vivo *of *columella cells*;

*(ii) *The next cell is rarely dividing and adopts the global maximum of auxin concentration. Its characteristics match those of the *root cap initials*;

*(iii) *The next cell is almost nondividing and corresponds in its characteristics to the *QC*;

*(iv) *The next cells in the *in silico *root are rarely dividing cells with a low auxin concentration. These characteristics are typical of the *vascular initials*;

*(v) *The actively dividing cells with a low auxin concentration correspond to the *meristematic zone *of RAM; and

*(vi) *Finally, the nondividing cells with a low auxin concentration correspond to the *differentiation zone *of the vascular system.

Thus, the positional information established *in silico *in the cells by the distributions of auxin and *Division Factor *forms characteristics that match cell fate specifications along the central axis in *A. thaliana *roots. The possible candidates for the role of *Division Factor *are considered in the Discussion.

### Simulation of Auxin Distribution under Various Conditions

The model analysis has demonstrated that the distal auxin maximum, found in the fifth cell of provascular layers, is formed for different sets of parameters and the model tolerates their slight variation. However, more significant changes in the parameter values led to a shift in the maximum position. Moreover, additional maxima of auxin concentration appear along the root axis [Additional files 1: Text S4; 7; 8]. In this section, we analyze these changes and give their biological interpretation.

#### Increasing auxin flow from the shoot results in formation of additional inner maxima

Under favorable conditions, the acropetal auxin flow from the aboveground part of developing plant constantly increases [39,40]. In the 1*D extended model*, the intensity of auxin flow from the shoot (*V*_*α*_) changes exogenously in time as *V*_*α *_= *α*_0 _+ *kt *[Additional file 1: Text S2]. If the value of *k *is small enough, the growth in *V*_*α *_is insufficient to compensate for the dilution of auxin concentration in the root caused by its growth (which is reached through increase in cell size and number). Consequently, the total auxin concentration in the root decreases, thereby leading first to the shift of the distal auxin maximum to the root end, , and then to complete disappearance of this maximum and formation of an approximately uniform concentration profile along the root, *a*_1 _≈ *a*_2 _≈... ≈*a*_*N*_. On the other hand, if a high growth in the auxin flow from the shoot to the root is specified, then the total auxin content in the *in silico *growing root will increase with time, thereby leading to periodic formation of additional auxin maxima near the first one (Figure 4D-F), [Additional file 9].

**Figure 5:**
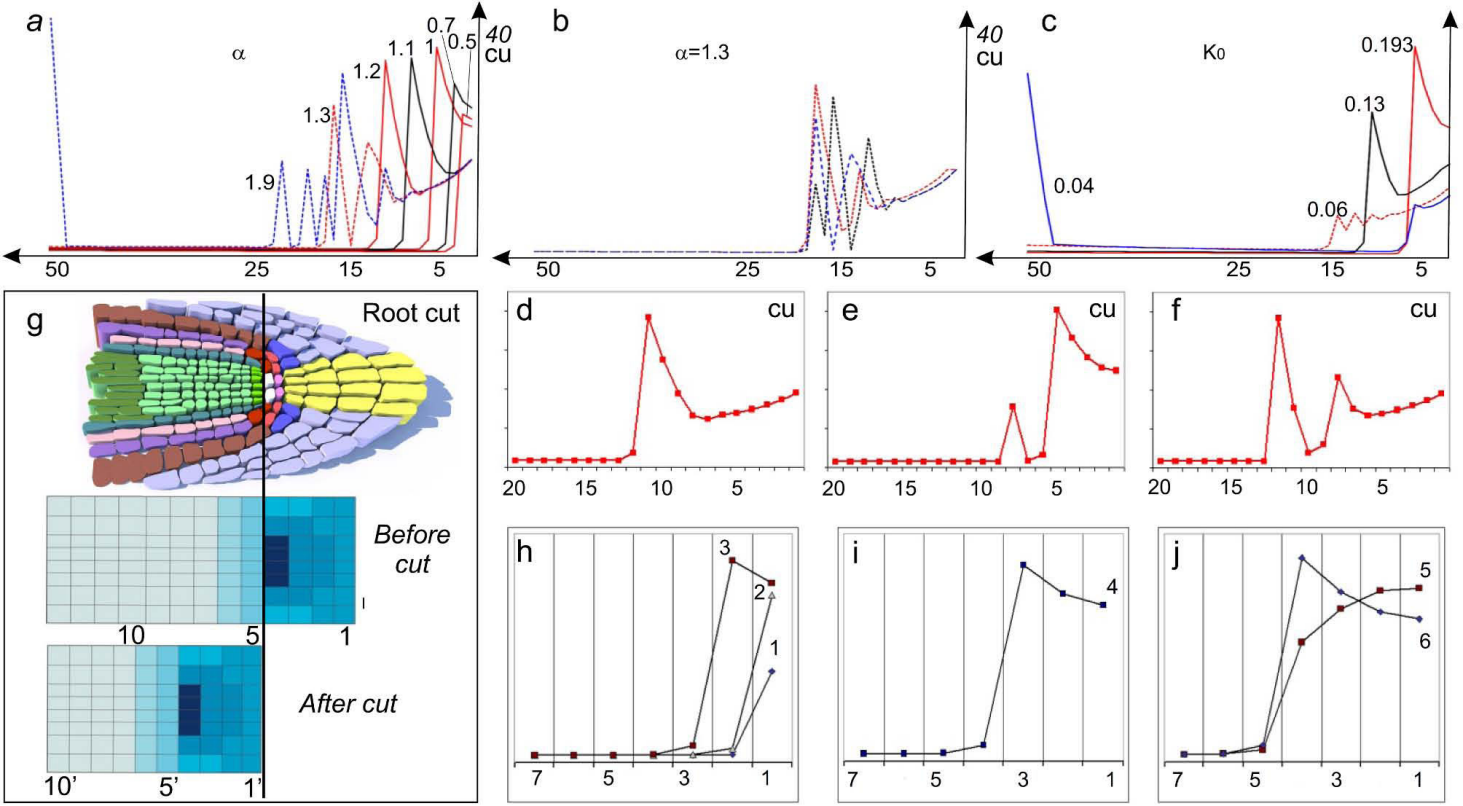
**Sensitivity of the auxin distribution pattern to parameter and initial data variations**. a.-c. The 1*D minimal model *analysis to variations of parameters *α *(a.-b.) and K_0 _(c.) that model changes in auxin flow from the shoot and treatment with auxin transport inhibitors, respectively. Oscillatory solutions of the model marked by dashed lines. b. When auxin flow from the shoot is too high, we observe unstable fluctuations of auxin concentration in the middle of the root. The curves correspond to unstable solutions calculated at equal time intervals. d.-f. The 1D *extended model *behavior under an increase in the auxin flow from the shoot when simulating the root growth. g.-j. Simulation of the experiment on QC laser ablation or root tip cut. g. Scheme of the *in silico *experiment on root tip cut. A qualitative correspondence of the model solutions to the auxin distribution pattern before and after the cut are shown. h.-j. Computer simulation of the changes in auxin distribution after the cut for the first seven cells. The curves are calculated for the provascular layer *j *= 4 and are numbered in the order the model solutions were obtained as the 2*D minimal model *approached the stationary state. In all plots, the *x *axis shows the cell number and the *y *axis, auxin concentration in concentration units (cu).

We used the 1*D minimal model *for a more detailed numerical study of the mechanisms leading to formation of multiple auxin maxima [Additional file 1: Text S4]. In this *in silico *experiment, we specified the root length as *N *= 50 cells and used the basic set of parameters, where the value of the rate *V*_*α *_was decreased to 0.01 *cu/tu*. We calculated the stationary auxin distribution by solving the Cauchy problem with zero initial data. Then using the parameter continuation method [41], we studied the evolution of this distribution with an increase in the *V*_*α*_. rate. At *V*_*α *_= 0.45, an auxin concentration maximum in the stationary solution was formed in the cell 2 and the subsequent increase in *V*_*α *_determined the shift of this maximum towards the root base. In particular, the distribution formed at *V*_*α *_= 1 fits the experimental distribution (Figure 4C), while at *V*_*α *_= 1.2, the maximum appears in cell 11 (Figure 5A). The *V*_*α *_rate had a critical value, *V*_*α *_~ 1.23, at which the stationary solution lost its stability. As a result, continuous oscillations of intracellular auxin concentrations appear in the model with a high-amplitude zone in the middle of the root (Figure 5A, B). Further increase in *V*_*α *_value (*α *> 1.75) results in formation of an auxin maximum at the root base in addition to fluctuating inner maxima. The oscillatory solutions found in the *1D minimal model *are replaced in the *1 D extended model *with solutions displaying periodic formation of additional auxin maxima. These maxima match the experimentally observed oscillation of auxin concentrations in the basal meristem that precede lateral root initiation [4]. The auxin maximum at the root base formed *in silico *under varying auxin flow from the shoot, may predetermine *in vivo *adventitious root initiation [5] which occurs rarely in *Arabidopsis*. The same calculations with the robust set of parameters showed that the additional auxin maximum forms in the stationary solutions by splitting of the proximally shifted auxin maximum into two separated maxima.

#### The effect of active auxin transport inhibitors on the auxin distribution pattern in the root

Treatment of the root with auxin active transport inhibitors, such as NPA or TIBA, leads to smearing of the auxin distribution pattern in the RAM [3]. Treatment with NPA shifts the auxin maximum towards the root base and decreases the maximum. The action of active transport inhibitors was simulated by decreasing the value of the constant of active transport rate *K_0 _*in the *1 D minimal model*. When calculating 1*D minimal model *with a moderately (no more than twofold) decreased *K_0 _*value, we observed a shift of the distal auxin maximum from the root tip towards its middle and a decrease in this maximum, which fit the experimental data. In addition, several low amplitude auxin concentration maxima appeared at *K_0 _*= 0.08-0.05 (Figure 5C). These peaks have not been described in experiments. Presumably, they were not recognized because of a small increase in auxin concentration (within the experimental error) in these peaks as compared with the neighboring cells but rather were interpreted as a weak uniform staining of the central root cylinder [3]. In the case of a considerable decrease in *K_0 _*value (less than 0.05), stationary solutions with two auxin maxima at the root base and at the root tip were formed (Figure 5C), which agrees well with the experimental data on high or prolonged NPA treatment [3]. We observed the same changes when we varied *q_3_*, the threshold of auxin-dependent PIN1 degradation [Additional files 1: Text S4; 7].

#### Self-restoration of auxin maximum and RAM structure after root tip cutting or QC ablation

After cutting off the root tip or ablating the QC by laser, the RAM gradually returns to its normal structure by forming a new QC from vascular initials [3,21,42]. We simulated these experiments *in silico *in the following manner. First, we calculated the stationary auxin concentration *a_j, i _*in the 2*D minimal model *at *M *= 8, *N *= 50 and the basic set of parameters. Then we took the cell layout of the 2*D minimal model *at *N *= 45 and simulated it with the initial data , *i *= 1...45. This configuration of the model with the specified distribution of auxin concentration simulates cutting off the root cap or QC ablation (leading to degeneration of the root cap) preserving intact the cells proximal to the QC (Figure 5G). Comparison of the *in silico *and *in vivo *experiments on regeneration of the distal auxin maximum demonstrated qualitative agreement of the changes in *PIN1 *and *DR5 *expressions, detected by Xu et al. (2006) [42], with the changes in PIN1 and auxin concentrations calculated in this model (Figure 5H-J), respectively. In particular, increases in the *DR5 *activity *in vivo *and auxin concentration *in silico *in the cells neighboring the destroyed QC (, *j *∈ {3, *M *-2}) were observed immediately after "QC ablation" in the corresponding experiments (Figure 5H, curves *1 *and *2*). In the model, this increase continued until the auxin concentration in the second cell from the ablated QC exceeded the threshold level , *j *∈ {3, *M *-2}), when an increased PIN1 degradation commenced; in this case, a new auxin maximum was formed in cell 2 by the *reflected flow *mechanism (Figure 5H, curve *3*). In the *in vivo *experiments, the formation of the new DR5 activity maximum near the ablated QC was also connected with a decrease in *PIN1 *expression in first and second cells from the ablated QC on the *x *axis [42]. The shift of the auxin maximum from the ablated QC in provascular layers continued with time *in silico *until a balance was reached between acropetal and reflected auxin flows (Figure 5I, J). The re-establishment of the stationary position of the auxin maximum and the restoration of the RAM structure around it were observed during 3 days after QC ablation [42]. In addition to generation of auxin maximum in the cells located proximally of the ablated QC (Figure 5G), in the 1D *extended model *we also observed *in silico *restoration of the specific characteristics of the cells (concentration of auxin and the rate of division) around the forming maximum, which suggest the regeneration of the overall RAM structure.

#### The auxin transport system can buffer changes in auxin concentration in localized tissues

IAA treatment of *A. thaliana *seedlings does not qualitatively change the auxin distribution pattern in the root. This suggests the presence of a homeostatic mechanism that maintains auxin distribution [12]. Substantial changes in the phenotype of *A. thaliana *root are observable only after treatment with high doses of exogenous auxin [4]. This type of experiment was studied *in silico *by changing the auxin concentration *a_i _*values in a cell, in the initial data of 1*D minimal model *[Additional file 1: Text S4]. Analysis of the calculations has demonstrated that auxin distribution pattern tolerates fluctuations less than 4 *cu*. The only effect of fluctuations above 4 *cu *is the shift in the auxin maximum position to the middle part of the root, , 6 ≤ *l *≤ 11. In the 1*D extended model*, the shift of the auxin maximum may be compensated by increasing the number of cells in the array so that the auxin maximum position maintains. Correspondingly, we inferred that the regulation mechanism for acropetal auxin transport described in the model at the basic set of parameter values provides resistance of the auxin distribution pattern to a stochastic variation of auxin concentrations in the cells, which agrees with experimental observations [12].

#### Different sets of parameter values can be realized in different plant species

The mechanisms of auxin transport in higher plants are highly conserved [43], yet the root architectures governed by auxin differ dramatically. There are two main types of root system: taproot and fibrous. It can be hypothesized that the *1D extended model *with different sets of parameters will describe the auxin distribution in the roots of different plant species, reflecting both the common and individual characteristics of distinct transport systems. It is of undoubted interest from this standpoint to study the *1D extended model *at different sets of parameters. Supplementary [Additional files 1: Text S4.2; 7, 8, 10] describes the analysis of behavior of the *1D extended model *with the "robust set" of parameters [Additional file 4: IV] which also gives a stationary solution that matches the experimental data [3]. We called this parameter set the "robust set", because the Hill coefficient *h*_2 _= 10 specifies a pronounced inhibition of the PIN1 expression when intracellular auxin concentration reaches the threshold value *q_3 _*= 3.26. In the 1*D extended model *with the robust set of parameters, the maximum of auxin concentration is maintained during root development. Yet the position of the distal maximum does not occupy a stringently constant position, "floating" in a certain range  where 10 ≤ *k *≤ 25. Characteristic of the model with the robust parameter set is also a greater diversity of stationary auxin distributions in the root, which display additional inner auxin maxima [Additional file 10] [44]. The auxin maxima emerge in response to increase in auxin flow from the shoot, to changes in initial data and to decrease of auxin active transport [Additional file 8]. Comparison of the models with robust and basic sets of parameters allows us to suggest potential distinctions between the systems of auxin transport that provide for formation of the fibrous and taproot systems (see Discussion).

## Discussion

In this work, we propose and substantiate a plausible mechanism for self-organization of the auxin distribution pattern along the central root axis, and its effect on establishment of the RAM. The *reflected flow *mechanism is based on the auxin-dependent regulation of auxin acropetal flow (Figure 1B): low auxin concentrations activate the transcription of *PIN *genes, whereas the high concentrations induce degradation of PIN proteins [24]. To test this mechanism against published experimental data, we created three versions of mathematical model (see the Methods section). The 1*D minimal model *simulates auxin distribution along a line of functionally identical non-dividing cells (Figure 1A). The 1*D extended model *expands the *1D minimal model *with rules of cell growth and division. In the 2*D minimal model *auxin moves in the rectangular cell layout of non-dividing cells that corresponds to a longitudinal cut of a three-dimensional root at early developmental stages (Figure 2A). In numerical experiments we showed that the *reflected flow *mechanism accounts for: (1) auxin distribution pattern formation in the root tip with the maximum at a certain distance form the root end; (2) maintenance of the auxin maximum in growing root; and (3) restoration of the auxin distribution pattern after the RAM damages. The models adequately reproduced experiments on root treatment by auxin transport inhibitors, exogenous auxin or after QC laser ablation. In simulation results we observed an additional effect of the dual dose-dependent regulation of PIN1 expression by auxin: increase of auxin flow from the shoot results in additional auxin maxima formation at the inner root cells or at the root base. We now discuss the biological impact of the simulation results and a set of testable predictions.

### The mechanisms of auxin distribution pattern formation

At least four main mechanisms of auxin distribution pattern formation have been suggested. The *flux-based polarization mechanism *relies on the canalization hypothesis proposed by Sachs [45] and describes a positive feedback between the auxin flux and the cell membrane permeability to auxin. The mechanism was first implemented in a mathematical model by Mitchison [46] and by Goldsmith et al. (1981) [47]. These models were extended for investigation of vein pattern formation [48,49] and for auxin distribution in plant meristems [17]. The *concentration-based polarization *mechanism provides for auxin distribution in tissue where the auxin flux towards the neighboring cell depends on the auxin concentration in the neighboring cell [13,14]. The *concentration-based polarization *mechanism was used for explanation of auxin pattern formation in the shoot apical meristem [13,14,50] as well as for vein pattern formation [51]. The third type of mechanism is the family of *structural mechanisms*, which imply that tissue structure determines the auxin distribution. The mechanism was first implemented by Grieneisen et al., (2007) [17], who studied the *reverse fountain *concept [11] in processes of auxin maximum formation in the root tip. A *structural mechanism *also has also been used to explain of auxin maxima formation in curved root regions [19].

Another well-known mechanism that can describe auxin distribution patterning but hasn't been implemented for this purpose before is the *activator-inhibitor *mechanism. The *activator-inhibitor *mechanism explains morphogenetic pattern formation under a positive and a negative regulation. This mechanism was first implemented in the reaction-diffusion model by Turing [52] and then extended by Meinhard [53]. These works initiated the mathematical theory of patterning processes in biology but haven't found an application in plant root molecular biology. Here we propose a possible mechanism for pattern formation by the distribution of auxin in root that originates in an *activator-inhibitor *mechanism. In the *reflected flow *mechanism presented here, auxin is both the activator and the inhibitor of the expression of its carrier (PIN1). The results described in the present work demonstrate that the dual regulation of polar auxin transport is a sufficient condition for self-organizing and maintenance of auxin distribution pattern in root tip during plant development.

### The Reverse Fountain Mechanism vs The Reflected Flow Mechanism

In this study we have shown that the *reflected flow *mechanism is a plausible alternative to the *reverse fountain *mechanism for auxin pattern formation in the root tip. The model of Grieneisen et al. (2007) [17] that implements the *reverse fountain *mechanism explains the generation of the auxin distribution pattern in the root tip based on a specific RAM structure in which each cell has a specified set of directions of auxin efflux (Figure 2B). In the model, both the level and direction polarization of PIN proteins in each cell are fixed. The *reflected flow *mechanism likewise doesn't consider the processes of PIN protein polarization, but unlike the *reverse fountain *mechanism, incorporates molecular processes of auxin regulation of the level of any PIN proteins present. Both the *reverse fountain *mechanism and the *reflected flow *mechanism adequately reproduce the distal auxin maximum formation in the root tip. However, these mechanisms differ in key respects.

There are at least three effects provided exclusively by the *reverse fountain *mechanism that shows its advantages over the *reflected flow *mechanism. First, the auxin pattern generated under the *reverse fountain *mechanism is extremely robust to the parameters variation whereas in our simulations are sensitive to changes in parameters value [Additional files 7; 8]. Second, the *reverse fountain *mechanism provides for formation of the gradient from the distal auxin maximum towards the root base, whereas the *reflected flow *mechanism does not. Third, the model [17] reproduces the experiments on auxin pattern maintenance in the root tip after root cut from the shoot, however the present model does not. All these advantages arise from specific features of the structural layout. Thus, *reverse fountain *mechanism more accurately explains the auxin distribution pattern in a mature root, where the structural layout is already formed.

The main advantage of the *reflected flow *mechanism over the *reverse fountain *mechanism becomes evident by comparison of the *in silico *experiments on root tip cut or QC ablation. In the model [17], simulation of disruption of the cell layout, as in the case of damage of the RAM structure after QC ablation, resulted for this model in auxin accumulation only in the first cell from the ablated QC; and the initial auxin distribution pattern of the intact root was not restored. This is in contrast to *in vivo *observations, where the restoration of the auxin maximum at a distance of the ablated QC precedes the restoration of both the QC and overall RAM structure [42]. In our simulation of root tip cut in the 2*D minimal model *we observed restoration of the auxin maximum at a distance from the new root end that matches experimental data [42] (Figure 5G). Moreover, the changes in auxin and PIN1 levels in our *in silico *experiment reproduced step-by-step the changes in DR5 and PIN1 expressions observed *in vivo *in course of RAM regeneration [42] (Figure 5H-J). Therefore, the *reflected flow *mechanism provides a better explanation of auxin pattern formation in developing RAM or RAM recovering after damage. In addition, unlike the prespecified RAM structure with five types of cells assumed in the model [17], the same auxin pattern was generated in the *2 D minimal model *with the only two cell types (Figure 2A, B). So the *reflected flow *mechanism describes the early steps in root development when cells are not specialized, and the concentration gradients play the primary role in their differentiation by providing positional information for their specification.

By comparison with the *reverse fountain *mechanism, the *reflected flow *mechanism is less robust to parameter variation. Thus, the *reflected flow *mechanism may contribute to root system response to environmental changes. The model analysis showed that the variations in values of some parameters provide reasonable explanations for changes in auxin distribution pattern (1) after root treatment with auxin and its analogs, (2) in response to increase of auxin flow from the shoot while plant is growing, or (3) to fluctuations in auxin flow caused by circadian rhythms.

We suggest that the *reverse fountain *and the *reflected flow *mechanisms are complementary. In particular, the *reflected flow *mechanism commences operating from the very early stages of root development. At later developmental stages, an anatomical structure forms and provides for the functioning of the *reverse fountain *mechanism that serve for more robust maintenance of the auxin maximum in the RAM. However, the *reflected flow *mechanism does not disappear even after formation of the RAM structure. Its role becomes less evident in the background of the structural mechanism of the *reverse fountain*, but morphogenes continue to function, revealing themselves if structure is disrupted or the environment changes.

### A Plausible Mechanism of Rhizotaxis

During plant growth, the acropetal auxin flow from the aboveground plant part to the root increases during development and plays a key role in regulating elongation of the main root and development of lateral roots [39,40]. Increase in auxin flow from the shoot occurs due to plant growth [40] and also daily due to circadian fluctuations in the auxin flow [54]. Analysis of our *in silico *experiments demonstrated that upon increase in auxin flow from the shoot to root an additional maximum was formed periodically in the provascular cells along with the distal auxin maximum [Additional file 9]. De Smet et al. (2007) [4] have demonstrated that the specification of the precursor cell for lateral root initiation can be first detected as early as in the protoxylem of the basal meristem where it correlates with an increase in auxin level. The local auxin concentration oscillates in the cells of the basal meristem at a regular period of 15 hours. Thus, regular fluctuations of the acropetal auxin flow according to circadian rhythms can be responsible for a rhizotactic pattern whereby under favorable conditions the next lateral root is initiated in regular time intervals and at certain distances from one another.

### How Acropetal Auxin Flow Determines Cell Fate Specification Along The Central Root Axis

In the 1*D extended model *cell divisions are regulated by auxin and a hypothetical morphogen *Division Factor*. The *Division Factor *combines the functions of cytokinin and ethylene in regulation of cell division rates in root - inhibition and activation, respectively (Figure 3E). For the profile of cell mitotic activity along the central axis of the root to be compatible with the experimental data (Figure 3A, B), we set a list of requirements for the mechanisms of *Division Factor *distribution (see the Methods section). The synthesis and degradation of the *Division Factor *depends on auxin (simulation with auxin as the only regulator of cell divisions failed to reproduce the experimental data; data not shown). The distributions of two morphogens form the positional information fields in the root patterning. In the model, we observed the appearance of individual cell characteristics, such as auxin concentration, relative localization on the axis, and mitotic activity, which could together correspond to the characteristics of various cell types located along the central root axis (Figure 3C).

The same profile of mitotic activity along the central root axis could be observed (data not shown) if we replace *Division Factor *by two agents: (1) a repressor of cell division, which is synthesized in the root cap and spreads over the root tissue from the root tip towards its base, forming a decreasing gradient (or which could be synthesized in all the root cells at a rate depending on auxin concentration), and (2) an activator of cell division, which is synthesized in the QC cells and anisotropically diffuses in root. The activator distribution matches to the domain of the ethylene precursor ACC expression [55], whereas the repressor distribution matches to the cytokinin [30] or auxin [3] distribution pattern. Thus, our simulation showed that the hormonal regulation of cell divisions in root may explain the root patterning.

### Simulation of Auxin Transport in Different Plant Species

Auxin transport in plants is a highly conserved mechanism: auxin distribution patterns with the maximum in the stem cells of the RAM have been demonstrated for *A. thaliana *[3], rice [56], and maize [57]. The core mechanism of auxin transport regulation, described in the model, may also be common for all higher plants. In particular, the role of PIN proteins in the auxin distribution in the root was demonstrated for *A. thaliana *[7], rice [58], and maize [59]. Thus, specific features of the auxin transport systems from different plants can be studied *in silico *by varying the parameters of the model. Both the "basic" and "robust" sets of parameters provide for generating and maintaining the distal auxin maximum, although the detailed model behavior differed depending on which set was used [Additional files 1: Text S4; 10].

First, the model with the robust set of parameters is sensitive to auxin fluctuations--additional auxin concentration maxima appeared in the middle of the root and at its base in simulations of root treatment with rather low doses of exogenous auxin. Moreover, once formed, these additional maxima were stably retained during root growth in the 1*D extended model*. Second, additional auxin maxima arose in stationary solutions when we (1) increased auxin flow from the shoot, (2) decreased the rate of auxin transport; (3) modified the coefficients for auxin-dependent *PIN1 *expression [Additional files 1: Text S4; 8]. Finally, formation of additional auxin maxima at the root base was more frequent in response to varying parameter values in the robust set compared to the basic set. These maxima may underlie the mechanism of adventitious roots initiation. All these characteristics agree well with the corresponding observations on roots of cereals (for example, maize). A prominent feature of cereal root systems is an elevated ability to develop lateral roots; for maize it was shown that this ability increases with the concentration of exogenous auxin [60]. We also noticed the possible difference in size of the QC zone in the *in silico *growing roots under different sets of parameters [Additional file 1: Text S4].

Analysis of the model's behavior using two sets of parameters suggests a key role for the auxin transport inhibition mechanisms in the formation of different root system types. The model's behavior with the basic set (low efficiency of auxin-dependent inhibition of *PIN1 *expression) agrees more closely with the pattern of auxin transport in the taproot system, while the model with robust set of parameters (high inhibition efficiency) better simulates the transport system of fibrous roots [Additional file 10].

## Conclusions

In this work, we have studied *in silico *a plausible mechanism providing for the generation of the auxin distribution patterns in the root, and its role in root patterning. We have demonstrated that the *reflected flow *mechanism that relies on the presence of positive and negative regulations between auxin and expression of its carriers provides not only for self-organization of the observed auxin distribution in the root, but also can explain much of the positional information in root patterning. Further, the resulting auxin distribution can be subsequently fixed in place by the *reverse fountain *mechanism [17]. Consideration of both mechanisms in one model will enhance future studies into the processes involved in root system development, such as the changes in RAM anatomy from the embryo to senescence, initiation and development of the lateral roots, formation of the fibrous root system, and so on.

## Authors' contributions

VVM, EM, VAL and NAO wrote the manuscript. VVM carried out the majority of the model design, calculation, analyses and the results interpretation. NAO and NAK contributed to the design and planning of the simulations, participated in data analysis. GY created the model with cell dynamics and performed its analysis. SIF performed the model calculation and analysis. EM developed the model with cell dynamics, and contributed to the design and planning of the simulations. VAL developed the mathematical model, conceived of the study, participated in its design and data interpretation. All authors read and approved the final manuscript.

## Supplementary Material

Additional file 1 **The Models details**. The supplementary text containing the following chapters: *Text S1*. The 2*D minimal model *equation. *Text S2*. Dynamical Grammar formulation and its application to modeling of auxin distribution in growing root. *Text S3*. The model parameters. *Text S4*. The analysis of the 1*D minimal model *with different sets of parameters. Click here for file

Additional file 2 **The 1*D extended model *in Mathematica**. The Mathematica file implementing the 1*D extended model *described in the main text. Click here for file

Additional file 3 **Processing of the experimental data on auxin distribution in root**. The figure showing the applied method of the experimental data on auxin distribution from DR5 auxin response images conversion to relative auxin concentrations in root cells. In the figure, (a) auxin distribution in the root according to DR5 reporter activity from Sabatini et al. (1999) [3]; (b) The result of processing image (a) using the ImageJ program, the intensity of staining along an image slice corresponding to the central root axis; (c) presenting this plot with cell layout and (d) model solutions matching the auxin distribution from (a), (b) and (c). Click here for file

Additional file 4 **The model parameters**. The table containing all model parameters. Click here for file

Additional file 5 **Simulation of root growth along the central root axis**. In the figure, (a) Distribution of auxin (red), Y (blue) and rates of cell division (gray columns) in conventional units along the central root axis. The curves were calculated in the 1 D *minimal model *with basic set of parameters. (b-d) The extended model solutions: (b) The mitotic activity along the central root axis; (c-f). Auxin and substance Y distribution. The green curve indicates the growth mode of cells (1- idle, 0- growth). c. the model was started from three cells; d. 10 cells; e. 20 cells; (f.) more than 100 cells. Cells of different types can be distinguished by considering both auxin concentration in the cell and its mitotic activity (see the main text for more details): QC - quiescent center; RCI - root cap initial; RC- root cap; MZ- meristematic zone; DZ- differentiation zone. Click here for file

Additional file 6 **Maintenance of auxin maximum at the root tip in the *in silico *growing root under normal condition**. The movie 1 simulated using the 1D *extended model *[Additional file 10] with basic set of parameters and auxin flow from the shoot *α *= 0.3+1.7*10^-5^*t*, auxin (red line), substance Y (blue line), cell phase (green line, 0 -GP; 1-IP). Click here for file

Additional file 7 **Analysis of the 1*D minimal model *with basic set of parameters**. The figure showing changes in the auxin distribution pattern at N = 50 in response to variations in: (a-b). *α *value; (c). diffusion rate (*D*) value; (d). *K_0 _*value; (e). *q_1 _*value; (f). *q_2 _*value; (g). *q_3 _*value; (h). *Kd *value, where other parameters were defined as in [Additional file 2: II]. Auxin distribution pattern calculated with the basic set of parameters and matching experimental data is marked by asterisk. Unstable fluctuations of auxin concentration in time are marked by dashed lines. The distributions having additional auxin maxima at the root base are blue colored. (e-k) The 1 D *minimal model *analysis in the STEP+ package [37]. e. The stationary solutions for i^th ^cells obtained by the method of continuation with respect to parameter *α*. The number of crossings of the selected component with a vertical line at *α *= 1 corresponds to the total number of stationary solutions (stable and unstable), with the same set of parameters. k. The stationary solutions of the model estimated on figure (e.). j. Oscillation of auxin concentration in the i^th ^cells in time (*tu*). In all plots, the *y *axis specifies auxin concentration in concentration units (*cu*). Click here for file

Additional file 8 **Analysis of the 1*D minimal model *with robust set of parameters**. The figure showing changes in the auxin distribution pattern at N = 50 in response to variations in: a. *α *value; b.-c. diffusion rate (*D*) value; d. *K_0 _*value; e. *q_1 _*value; f. *q_2 _*value; g. *q_3 _*value; h. *Kd *value, where other parameters were defined as in [Additional file 2: IV]. Auxin distribution pattern calculated with the robust set of parameter values and matching experimental data is marked by asterisk. Unstable fluctuations of auxin concentration in time are marked by dashed lines. The distributions having additional auxin maxima at the root base are blue colored. e. The 1 D *minimal model *analysis with the robust set of parameters in the STEP+ package [37]. The stationary solutions for i^th ^cells are obtained by the method of continuation with respect to parameter *α*. The number of crossings (102) of the selected component with a vertical line at *α *= 1 corresponds to the total number of stationary solutions (stable and unstable), with the same set of parameters. In all plots, the *y *axis specifies auxin concentration in concentration units (*cu*). Click here for file

Additional file 9 **Periodic formation of additional inner auxin maximum in the *in silico *growing root under increased rates of auxin flow from the shoot**. The movie 2 simulated using the 1*D extended model *[Additional file 10] with robust set of parameters and auxin flow from the shoot *α *= 0.4+1.7*10^-5^*t*, auxin (red line), substance Y (blue line), cell phase (green line, 0 -GP; 1-IP). Click here for file

Additional file 10 **Comparison of the models behavior with basic and robust sets of parameters**. The table showing the differences in the *minimal *and *extended models *behavior with basic and robust sets of parameters. Click here for file

## References

[B1] Dolan L Janmaat K Willemsen V Linstead P Poethig S Roberts K Scheres B Cellular organisation of the Arabidopsis thaliana root Development 1993 119 71 84 8275865 10.1242/dev.119.1.71

[B2] De Smet I Jurgens G Patterning the axis in plants - auxin in control Curr Opin Genet Dev 2007 17 337 43 10.1016/j.gde.2007.04.012 17627808

[B3] Sabatini S Beis D Wolkenfelt H Murfett J Guilfoyle T Malamy J Benfey P Leyser O Bechtold N Weisbeek P Scheres B An Auxin-Dependent Distal Organizer of Pattern and Polarity in the *Arabidopsis *Root Cell 1999 99 5 463 472 10.1016/S0092-8674(00)81535-4 10589675

[B4] De Smet I Tetsumura T De Rybel B Frey NF Laplaze L Casimiro I Swarup R Naudts M Vanneste S Audenaert D Inzé D Bennett MJ Beeckman T Auxin-dependent regulation of lateral root positioning in the basal meristem of Arabidopsis Development 2007 134 4 681 690 10.1242/dev.02753 17215297

[B5] Gonzali S Novi G Loreti E Paolicchi F Poggi A Alpi A A turanose-insensitive mutant suggests a role for WOX5 in auxin homeostasis in Arabidopsis thaliana Plant J 2005 44 4 633 45 10.1111/j.1365-313X.2005.02555.x 16262712

[B6] Reinhardt D Pesce E Stieger P Mandel T Baltensperger K Bennett M Traas J Friml J Kuhlemeier C Regulation of phyllotaxis by polar auxin transport Nature 2003 426 6964 255 260 10.1038/nature02081 14628043

[B7] Blilou I Xu J Wildwater M Willemsen V Paponov I Friml J Heidstra R Aida M Palme K Scheres B The PIN auxin efflux facilitator network controls growth and patterning in Arabidopsis roots Nature 2005 433 7021 39 44 10.1038/nature03184 15635403

[B8] Benkova E Michniewicz M Sauer M Teichmann T Seifertova D Jurgens G Friml J Local, efflux-dependent auxin gradients as a common module for plant organ formation Cell 2003 115 5 591 602 10.1016/S0092-8674(03)00924-3 14651850

[B9] Vanneste S Friml J Auxin: a trigger for change in plant development Cell 2009 136 6 1005 1016 10.1016/j.cell.2009.03.001 19303845

[B10] Kramer EM Bennett MJ Auxin transport: a field in flux Trends Plant sci 2006 11 8 382 386 10.1016/j.tplants.2006.06.002 16839804

[B11] Swarup R Benett M Auxin Transport: The Fountain of Life in Plants? Developmental Cell 2003 5 6 824 826 10.1016/S1534-5807(03)00370-8 14667404

[B12] Friml J Benkova E Blilou I Wisniewska J Hamann T Ljung K Woody S Sandberg G Scheres B Jürgens G Palme K AtPIN4 mediates sink-driven auxin gradients and root patterning in Arabidopsis Cell 2002 108 5 661 73 10.1016/S0092-8674(02)00656-6 11893337

[B13] Jönsson J Heisler Mg Shapiro BE Meyerowitz EM Mjolsness E An auxin-driven polarized transport model for phyllotaxis Proc Natl Acad Sci USA 2006 103 5 1633 1638 10.1073/pnas.0509839103 16415160 PMC1326488

[B14] Smith RS Guyomarch S Mandel T Reinhardt D Kuhlemeier C Prusinkiewicz P A plausible model of phyllotaxis Proc Natl Acad Sci USA 2006 103 5 1301 1306 10.1073/pnas.0510457103 16432192 PMC1345713

[B15] de Reuille PB Bohn-Courseau I Ljung K Morin H Carraro N Godin C Traas J Computer simulations reveal properties of the cell-cell signaling network at the shoot apex in Arabidopsis Proc Natl Acad Sci USA 2006 103 5 1627 1632 10.1073/pnas.0510130103 16432202 PMC1360567

[B16] Stoma S Lucas M Chopard J Schaedel M Traas J Godin C Flux-Based Transport Enhancement as a Plausible Unifying Mechanism for Auxin Transport in Meristem Development PLOS Comp biol 2008 4 10 e1000207 10.1371/journal.pcbi.1000207 PMC2565506 18974825

[B17] Grieneisen VA Xu J Marée AF Hogeweg P Scheres B Auxin transport is sufficient to generate a maximum and gradient guiding root growth Nature 2007 449 7165 1008 1013 10.1038/nature06215 17960234

[B18] Doerner P Plant roots: recycled auxin energizes patterning and growth Curr Biol 2008 18 2 R72 74 10.1016/j.cub.2007.11.048 18211844

[B19] Laskowski M Grieneisen VA Hofhuis H Hove CA Hogeweg P Marée AF Scheres B Root system architecture from coupling cell shape to auxin transport PLoS Biol 2008 6 12 e307 10.1371/journal.pbio.0060307 19090618 PMC2602721

[B20] Friml J Vieten A Sauer M Weijers D Schwarz H Hamann T Offringa R Jürgens G Efflux-dependent auxin gradients establish the apical-basal axis of Arabidopsis Nature 2003 426 6963 147 153 10.1038/nature02085 14614497

[B21] van den Berg C Willemsen V Hage W Weisbeek P Scheres B Cell fate in the Arabidopsis root meristem determined by directional signalling Nature 1995 378 6552 62 65 10.1038/378062a0 7477287

[B22] Benková E Ivanchenko MG Friml J Shishkova S Dubrovsky JG A morphogenetic trigger: is there an emerging concept in plant developmental biology? Trends Plant Sci 2009 14 4 189 193 10.1016/j.tplants.2009.01.006 19285906

[B23] Prusinkiewicz P Crawford S Smith RS Ljung K Bennett T Ongaro V Leyser O Control of bud activation by an auxin transport switch Proc Natl Acad Sci USA 2009 106 41 17431 17436 10.1073/pnas.0906696106 19805140 PMC2751654

[B24] Vieten A Vanneste S Wisniewska J Benkova E Benjamins R Beeckman T Luschnig C Friml J Functional redundancy of PIN proteins is accompanied by auxin dependent cross-regulation of PIN expression Development 2005 132 20 4521 4531 10.1242/dev.02027 16192309

[B25] Sauer M Balla J Luschnig C Wisniewska J Reinöhl V Friml J Benková E Canalization of auxin flow by Aux/IAA-ARF-dependent feedback regulation of PIN polarity Genes & Dev 2006 20 2902 2911 10.1101/gad.390806 PMC1619939 17043314

[B26] Ljung K Hull AK Celenza J Yamada M Estelle M Normanly J Sandberg G Sites and regulation of auxin biosynthesis in Arabidopsis roots Plant Cell 2005 17 4 1090 1104 10.1105/tpc.104.029272 15772288 PMC1087988

[B27] Beemster GT Baskin TI Stunted plant 1 mediates effects of cytokinin, but not of auxin, on cell division and expansion in the root of Arabidopsis Plant Physiol 2000 124 4 1718 1727 10.1104/pp.124.4.1718 11115888 PMC59869

[B28] Campanoni P Nick P Auxin-dependent cell division and cell elongation. 1-Naphthaleneacetic acid and 2,4-dichlorophenoxyacetic acid activate different pathways Plant Physiol 2005 137 3 939 948 10.1104/pp.104.053843 15734918 PMC1065395

[B29] Ortega-Martínez O Pernas M Carol RJ Dolan L Ethylene modulates stem cell division in the Arabidopsis thaliana root Science 2007 317 5837 507 510 10.1126/science.1143409 17656722

[B30] Dello Ioio R Linhares FS Scacchi E Casamitjana-Martinez E Heidstra R Costantino P Sabatini S Cytokinins determine Arabidopsis root-meristem size by controlling cell differentiation Curr Biol 2007 17 8 678 682 10.1016/j.cub.2007.02.047 17363254

[B31] Mjolsness E Yosiphon G Stochastic Process Semantics for Dynamical Grammars Ann Math Artif Intell 2006 47 329 395 10.1007/s10472-006-9034-1

[B32] Stochastic Parameterized Grammars: Formalization, Inference and Modeling Applications 2009 PhD thesis, University of California Irvine

[B33] Yosiphon G PhD thesis http://computableplant.ics.uci.edu/~guy/downloads/papers/thesis.pdf

[B34] Likhoshvai V Ratushny A Generalized hill function method for modeling molecular processes J Bioinform Comput Biol 2007 5 2B 521 531 10.1142/S0219720007002837 17636859

[B35] Kazantsev FV Akberdin IR Bezmaternykh KD Lashin SA Podkolodnaya NN Likhoshvai VA Kolchanov NA, Ralf Hofestadt A computer system for reconstruction, calculation and analysis of mathematical models of molecular genetic systemproceedings of the Sixth Intern. Conf. on Bioinformatics of Genome Regulation and Structure (BGRS'2008): 22-28 June 2008; Novosibirsk, Russia. Novosibirsk 2008 113

[B36] Gear CW The Automatic Integration of Ordinary Differential Equations Comm Ass Comput Mach 1971 14 1 176 190

[B37] Fadeev SI Korolev VK Gainova IA Medvedev AE The package STEP+ for numerical study of autonomous systems arising when modeling dynamics of genetic-molecular systems Proceeding of the Sixth International Conference on Bioinformatics of Genome Regulation and Structure: 16-22 July 2006; Novosibirsk. V. 3.2 118

[B38] Collins TJ ImageJ for microscopy Biotechniques 2007 43 Suppl 1 25 30 10.2144/000112517 17936939

[B39] Reed RC Brady SR Muday GK Inhibition of auxin movement from the shoot into the root inhibits lateral root development in Arabidopsis Plant Physiol 1998 118 4 1369 1378 10.1104/pp.118.4.1369 9847111 PMC34753

[B40] Lovin MB Buer CS Muday GK Environmental modulation of root branching by changing auxin flow from the shootproceedings of the 4th International Symposium on Adventitious Root Formation 2004 Savannah, GA USA 5

[B41] Kogai VV Fadeev SI Application of parameter continuation based on the multiple shooting method for the numerical investigation of nonlinear boundary value problems Sib Zh Ind Mat 2001 4 83 101

[B42] Xu J Hofhuis H Heidstra R Sauer M Friml J Scheres B A molecular framework for plant regeneration Science 2006 311 5759 385 388 10.1126/science.1121790 16424342

[B43] Cooke TJ Poli DB Sztein AE Cohen JD Evolutionary patterns in auxin action Plant Molecular Biology 2002 49 319 338 10.1023/A:1015242627321 12036257

[B44] Likhoshvai VA Omel'ianchuk NA Mironova VV Fadeev SI Mjolsness ED Kolchanov NA Mathematical model of auxin distribution in the plant root Russian journal of developmental biology 2007 38 446 456 10.1134/S1062360407060057 18179024

[B45] Sachs T Polarity and the induction of organized vascular tissues Ann Bot (Lond) 1969 33 263

[B46] Mitchison GJ A model for vein formation in higher plants Proc R Soc Lond B 1980 207 79 109 10.1098/rspb.1980.0015

[B47] Goldsmith MHM Goldsmith TH Martin MH Mathematical analysis of the chemosmotic polar diffusion of auxin through plant tissues Proc Natl Acad Sci USA 1981 78 2 976 980 10.1073/pnas.78.2.976 16592983 PMC319928

[B48] Feugier FG Iwasa Y How canalization can make loops: a new model of reticulated leaf vascular pattern formation J Theor Biol 2006 243 2 235 44 10.1016/j.jtbi.2006.05.022 16887150

[B49] Bayer EM Smith RS Mandel T Nakayama N Sauer M Prusinkiewicz P Kuhlemeier C Integration of transport-based models for phyllotaxis and midvein formation Genes Dev 2009 23 3 373 384 10.1101/gad.497009 19204121 PMC2648550

[B50] Sahlin P Söderberg B Jönsson H Regulated transport as a mechanism for pattern generation: capabilities for phyllotaxis and beyond J Theor Biol 2009 258 1 60 70 10.1016/j.jtbi.2009.01.019 19490869

[B51] Merks RM Van de Peer Y Inzé D Beemster GT Canalization without flux sensors: a traveling-wave hypothesis Trends Plant Sci 2007 12 9 384 390 10.1016/j.tplants.2007.08.004 17765595

[B52] Turing A The chemical basis of morphogenesis Phil Trans Roy (Lon) 1952 237 37 72 10.1098/rstb.1952.0012

[B53] Meinhardt H Models of biological pattern formation 1982 London: Academic Press

[B54] Jouve L Gaspar T Kevers C Greppin H Degli Agosti R Involvement of indole-3-acetic acid in the circadian growth of the first internode of Arabidopsis Planta 1999 209 136 42 10.1007/s004250050615 10467040

[B55] Rodrigues-Pousada RA De Rycke R Dedonder A Van Caeneghem W Engler G Montagu MV Van Der Straetena D The Arabidopsis 1-Aminocyclopropane-1-Carboxylate Synthase Gene 1 Is Expressed during Early Development Plant Cell 1993 5 8 897 911 10.1105/tpc.5.8.897 12271088 PMC160325

[B56] Scarpella E Rueb S Meijer AH The RADICLELESS1 gene is required for vascular pattern formation in rice Development 2003 130 4 645 658 10.1242/dev.00243 12505996

[B57] Kerk NM Feldman LJ A biochemical model for the initiation and maintenance of the quiescent center: implications for organization of root meristems Development 1995 121 2825 2833

[B58] Xu M Zhu L Shou H Wu P A PIN1 family gene, OsPIN1, involved in auxin-dependent adventitious root emergence and tillering in rice Plant Cell Physiol 2005 46 1674 1681 10.1093/pcp/pci183 16085936

[B59] Carraro N Forestan C Canova S Traas J Varotto S ZmPIN1a and ZmPIN1b encode two novel putative candidates for polar auxin transport and plant architecture determination of maize Plant Physiol 2006 142 254 264 10.1104/pp.106.080119 16844839 PMC1557596

[B60] Kerk NM Jiang K Feldman LJ Auxin metabolism in the root apical meristem Plant Physiol 2000 122 925 932 10.1104/pp.122.3.925 10712557 PMC58929

