# Supplementary material for: A plausible mechanism for auxin patterning along the developing root
Source: BMC Syst Biol. 2010 Jul 21;4:98. doi: 10.1186/1752-0509-4-98 (PMC2921385; doi:10.1186/1752-0509-4-98)
Supplement: Additional file 1 — The Models details. The supplementary text containing the following chapters: Text S1. The 2D minimal model equation. Text S2. Dynamical Grammar formulation and its application to modeling of auxin distribution in growing root. Text S3. The model parameters. Text S4. The analysis of the 1D minimal model with different sets of parameters. [file 1752-0509-4-98-S1.PDF]

Supplementary text

To the paper

A plausible mechanism for auxin patterning along the developing root

Text S1. 2*D minimal model* equations

The cell layout of the 2D model is the rectangular that consists of *M* layers (*j=1,…,M*) of *N* cells each (*i=1…N*). Two layer types are considered: provascular (*j=3,…,M-2)* and epidermal (*j=1,2…,M-1,M).* For the details, see the Method section in the main text. The auxin and PIN1 concentrations in the cell located in the *i*th cell of the *j*th layer are described by variables $a_{j,i}$ and $PIN_{j,i}$, respectively.

The Ordinary Differential Equations for the provascular layers are:

$$\begin{cases} \dfrac{da_{j,1}}{dt} = K_o\, a_{j,2} PIN_{j,1} + D\cdot(a_{j,2} + a_{j+1,1} + a_{j-1,1}) - 3D\cdot a_{j,1} - K_d a_{j,1} \\ \dfrac{dPIN_{j,1}}{dt} = k_1 \left(\dfrac{a_{j,1}}{q_1}\right)^{h_1} \Big/ \left(1+\left(\dfrac{a_{j,1}}{q_2}\right)^{h_1}\right) - k_2 PIN_{j,1}\left(1+\left(\dfrac{a_{j,1}}{q_3}\right)^{h_2}\right) \\ \ldots \\ \dfrac{da_{j,i}}{dt} = D(a_{j,i+1} + a_{j,i-1} + a_{j+1,i} + a_{j-1,i}) + K_o a_{j,i+1} PIN_{j,i+1} - 4Da_{j,i} - K_o a_{j,i} PIN_{j,i} - K_d\, a_{j,i} \\ \dfrac{dPIN_i}{dt} = k_1 \left(\dfrac{a_{j,i}}{q_1}\right)^{h_1} \Big/ \left(1+\left(\dfrac{a_{j,i}}{q_2}\right)^{h_1}\right) - k_2 PIN_{j,i}\left(1+\left(\dfrac{a_{j,i}}{q_3}\right)^{h_2}\right) \\ \ldots \\ \dfrac{da_{j,N}}{dt} = \alpha + D(a_{j,N-1} + a_{j+1,N} + a_{j-1,N}) - 3Da_{j,N} - K_o\, a_{j,N}\, PIN_{j,N} - K_d\, a_{j,N} \\ \dfrac{dPIN_{j,N}}{dt} = k_1 \left(\dfrac{a_{j,N}}{q_1}\right)^{h_1} \Big/ \left(1+\left(\dfrac{a_{j,N}}{q_2}\right)^{h_1}\right) - k_2 PIN_{j,N}\left(1+\left(\dfrac{a_{j,N}}{q_3}\right)^{h_2}\right) \end{cases} \quad (1)$$

The Ordinary Differential Equations for the epidermal layers are:

For layers $j \in \{2, M-1\}$:

$$\frac{da_{j,i}}{dt} = D(a_{j,i+1} + a_{j,i-1} + a_{j+1,i} + a_{j-1,i}) - 4Da_{j,i} - K_d a_{j,i} \qquad (2)$$

For layers $j \in \{1, M\}$:

$$\begin{cases} \frac{da_{j,1}}{dt} = D(a_{j,2} - a_{j,1}) + D(a_{j+\delta_j,1} - a_{j,1}) - K_d a_{j,1} \\ \ldots \\ \frac{da_{j,i}}{dt} = D(a_{j,i+1} + a_{j,i-1} - 2a_{1,i}) + D(a_{j+\delta_j,i} - a_{j,i}) - K_d a_{j,i} \\ \ldots \\ \frac{da_{j,N}}{dt} = D(a_{j,N-1} - a_{j,N}) + D(a_{j+\delta_j,N} - a_{j,N}) - K_d a_{j,N} \end{cases} \qquad (3)$$

where $\delta_1 = +1, \delta_M = -1$.

The equations (1),(2), (3) define the full system of ordinary equations of the 2D minimal model.

Text S2. Dynamical Grammar formulation and its application to modeling of auxin distribution in growing root.

For analysis of auxin distribution in an array of dividing cells we employed a Dynamical Grammar (DG) realized in the *Plenum* package [1-3] for the *Mathematica* system. DG provides a multiscale modeling framework in which a system can be comprised of continuous and discrete elements, and evolves as a stochastic process. The transition probability operator is defined by stochastic rules (specified by the *with* clause in a generalized reaction) with corresponding rate functions, and continuous rules (*solving* clause that contains differential equations), that act on groups of parameterized objects. Syntax, semantics and simulation algorithm for such hybrid systems is described in [1].

Using DG we reproduced the model of auxin distribution in root (*the minimal model*). The *Plenum* package was used to extend *the minimal model* with rules of cell growth and division (*the 1D extended model*) [2-3] [Additional file 2]. In the application, the objects are cells parameterized by size (r), location on the x axis (x), cell mode (m) and

concentrations of signaling molecules auxin (*a*) and hypothetic substance *Division Factor* (*DivF*):

$$Cell(x, r, m, a, DivF)$$

2.1. *The minimal model* in DG formalism

Description of auxin concentration dynamics in *the minimal model* (equations 1-7 in the Models section) was reduced to DG semantics:

Auxin flow from the shoot

$$\{c_N = Cell(x_N, r_N, m_N, a_N, DivF_N)\} \to \{c_N\}$$

$$\text{solving}\left\{\frac{da_N}{dt} = \alpha_{init} + \frac{0.17t}{CellCycleTime}\right\}$$

which is defined only for the Nth cell in the array. Auxin flow from the shoot is assumed to be the only source of auxin in root. In *the 1D extended model*, the intensity of auxin flow grows in time according to the average duration of cell cycle ($CellCycleTime$ =0.1*10^5 model iterations), and the initial value of auxin flow from the shoot for the cell array with three cells (N=3) is α_{init} = 0.4.

Auxin degradation

$$\{c_i = Cell(x_i, r_i, m_i, a_i, DivF_i)\} \to c_i$$

$$\text{solving}\left\{\frac{da_i}{dt} = -a_i(d + \frac{v(r_i)}{r_i}), \frac{dr}{dt} = v(r)\right\}$$

Auxin degradation combines processes of irreversible losses of auxin due to processes of conjugation, oxidation, auxin transport in other directions (not along *x*) and dilution ($v(r_i)/r_i$) due to cell growth. Degradation is defined for all cells in the array.

Auxin diffusion

$$\{c_i = Cell(x_i, r_i, m_i, a_i, DivF_i), \quad c_{i+1} = Cell(x_{i+1}, r_{i+1}, m_{i+1}, a_{i+1}, DivF_{i+1}), \\ s_{i,i+1} = spring(c_i, c_{i+1})\} \rightarrow \{c_i, c_{i+1}, s_{i,i+1}\}$$

$$\text{solving}\left\{\frac{da_i}{dt} = D(a_{i+1} - a_i), \frac{da_{i+1}}{dt} = D(a_i - a_{i+1})\right\}$$

An isotropic exchange of auxin between neighboring cells is defined in the model as linear in time diffusion. Coefficient of diffusion (*D*) is the same for all cells in the array (Table S1). Because of the boundary conditions, in the cells *i*=1 and *N* diffusion is defined only into (and from) the one neighboring cell (*i*=2 and *N-1*, respectively).

Active transport

$$\{c_i = Cell(x_i, r_i, m_i, a_i, DivF_i), \quad c_{i+1} = Cell(x_{i+1}, r_{i+1}, m_{i+1}, a_{i+1}, DivF_{i+1}), \\ s_{i,i+1} = spring(c_i, c_{i+1})\} \rightarrow \{c_i, c_{i+1}, s_{i,i+1}\}$$

$$\text{solving}\left\{\frac{da_{i+1}}{dt} = -K_0 a_{i+1} PIN(a_{i+1}), \frac{da_i}{dt} = K_0 a_{i+1} PIN(a_{i+1})\right\}$$

Active auxin transport in the $N \rightarrow 1$ direction (acropetal flow) is defined for all cells in the array except *i=1* due to the boundary condition. The rate of acropetal flow in *the minimal model* depends on concentration of PIN1 protein *(PIN*) in the current cell (Eq. 5-7 in the main text). On the other hand, rates of synthesis and degradation of PIN1 depends on the auxin concentration in the current cell:

$$\frac{dPIN_i}{dt} = k_1 \left(\frac{a_i}{q_1}\right)^{h_1} \Bigg/ \left(1 + \left(\frac{a_i}{q_2}\right)^{h_1}\right) - k_2 PIN_i \left(1 + \left(\frac{a_i}{q_3}\right)^{h_2}\right)$$

In *the 1D extended model*, we approximated the function of the rate of auxin active transport $PIN(a_i)$ by Tikhonov's theorem [4]. As a result, the number of ODE's was reduced twice, so that the rate of auxin active transport depends directly on the auxin concentration in the current cells:

$$PIN(a_i) = \frac{\left(\frac{a_i}{q_1}\right)^{h_1}}{1+\left(\frac{a_i}{q_2}\right)^{h_1}} \cdot \frac{1}{1+\left(\frac{a_i}{q_3}\right)^{h_2}}$$

Which define the function *PIN(a)* appearing in the active transport rule.

2.2. Modeling of cells growth and division under auxin regulation: *the 1D extended model*

a. Cell growth

Cells in the complex model can grow and divide. This dynamic behavior is modeled by DG rules that specify a weak (breakable) spring potential function between neighboring cells, as described in [5] and reviewed in [6]:

$$\{c_i = Cell(x_i, r_i, m_i, a_i, DivF_i),\ c_{i+1} = Cell(x_{i+1}, r_{i+1}, m_{i+1}, a_{i+1}, DivF_{i+1}),$$
$$s_{i,i+1} = spring(c_i, c_{i+1})\} \to \{c_i, c_{i+1}, s_{i,i+1}\}$$
$$\text{solving}\left\{\frac{dx_i}{dt} = \nabla V(d(x_i, x_{i+1}), r_i + r_{i+1})\right\}$$

where ∇V is a weak spring potential defined in [2].

In simulation of growth in the 1D array, cells are continually moving along the *x* axis by changing their size *r:*

$$Cell(x_i, r_i, m_i = 1, a_i, DivF_i) \to Cell(x_i, r_i, m_i = 1, a_i, DivF_i)$$
$$\text{solving}\left\{\frac{dr_i}{dt} = 10^{-5}\right\}$$

In the *1D extended model* cells have a discrete parameter *m* that denotes a cell's mode according to different phases of the cell cycle (Eqs. (9) and (10) in the main text). Two different modes of cell growth were considered: either *growth* (*m=1)* or *idle (m=2).* In the *growth* phase the cell is growing but can't divide regardless of auxin and *Division*

factor concentrations. Initially cells in the array are in the *growth* phase so *m=1*, but it changes with the size-dependent probability:

$$Cell(x_i, r_i, m_i = 1, a_i, DivF_i) \to Cell(x_i, r_i, m_i = 2, a_i, DivF_i)$$
$$\text{with } \rho_{GP}(r_i) = (1 + \exp(-(r_i - r_{\min})/T))^{-1},$$

where r_{min}=1 is normalized constant of cell size and tolerance parameter T=0.01.

In the *idle* phase, cells aren't growing but can divide with a probability that depends on the *Division Factor* concentration as will appear in section 1.2(b) below.

b. Hypothetical Division Factor

In the *1D extended model*, hypothetical *Division Factor* unites the functions of two agents: activator and inhibitor of cell division. *Division Factor* (*DivF*) is synthesized in cells depending on the difference in concentrations between neighboring cells (a_i with respect to a_{i+1}):

$$\{c_i = Cell(x_i, r_i, m_i, a_i, DivF_i),\ c_{i+1} = Cell(x_{i+1}, r_{i+1}, m_{i+1}, a_{i+1}, DivF_{i+1}),$$
$$s_{i,i+1} = spring(c_i, c_{i+1})\} \to \{c_i, c_{i+1}, s_{i,i+1}\}$$
$$\text{solving}\left\{\frac{dDivF_i}{dt} = \beta\left(1 + \exp\left(\frac{10 - (a_{i+1} - a_i)}{T}\right)\right)^{-1}\right\}$$

Due to the boundary condition, in the cell *i=N* synthesis of *Division Factor* is not defined. The sigmoid function $\sigma(a_{i+1} - a_i)$ describes the rate of *Division Factor* synthesis so that its value increases sharply when $a_{i+1} - a_i$ is over 10 concentration units, with tolerance parameter T=0.1. Thereby, *Division Factor* synthesizes mainly in the cell that is next to the one with the auxin maximum. *In vivo* this is the cell of the quiescent center.

Substance *Division Factor* diffuses along the cell array. The process of diffusion is described the same way as for auxin:

$$\{c_i = Cell(x_i, r_i, m_i, a_i, DivF_i), \quad c_{i+1} = Cell(x_{i+1}, r_{i+1}, m_{i+1}, a_{i+1}, DivF_{i+1}),$$
$$s_{i,i+1} = spring(c_i, c_{i+1})\} \to \{c_i, c_{i+1}, s_{i,i+1}\}$$
$$\text{solving}\left\{\frac{dDivF_i}{dt} = D_{DivF}(DivF_{i+1} - DivF_i), \frac{dDivF_{i+1}}{dt} = D_{DivF}(DivF_i - DivF_{i+1})\right\}$$

Where D_{DivF} is the diffusion rate constant for the *Division Factor*.

In *the 1D extended model*, *Division Factor* degradation is regulated by auxin in a dose-respondent manner. The rate of *Division Factor* degradation increases as a sigmoidal function of auxin concentration:

$$\{c_i = Cell(x_i, r_i, m_i, a_i, DivF_i)\} \to \{c_i\}$$
$$\text{solving}\left\{\frac{dDivF_i}{dt} = -DivF_i(K_{d,DivF}(a_i) + \frac{v(r_i)}{r_i})\right\}$$
$$K_{d,DivF}(a_i) = k^0_{d,DivF}\left(\left(1+\left(\frac{a_i}{k^1_{d,DivF}}\right)^{h_3}\right)\Bigg/\left(1+\left(\frac{a_i}{k^2_{d,DivF}}\right)^{h_4}\right)\right)$$

Where $k^0_{d,DivF}$ is the rate coefficient for auxin-dependent *Division Factor* degradation; $k^1_{d,DivF}$ is the threshold of auxin-dependent activation of *Division Factor* synthesis; $k^1_{d,DivF}$ is the threshold of auxin-dependent saturation of *Division Factor* synthesis; h_3 and h_4 are Hill coefficients which determine the response rate of these processes to the changes in intracellular auxin concentration. The *Division Factor* distribution pattern is generated as a result of foregoing processes. We exploit the pattern to simulate cells dynamics that qualitatively match the experimental data on cell divisions in root. There are two maxima of mitotic activity along the central root axis which are separated by the quiescent center (see the main text for details).

c. Cell division

In *the 1D extended model*, the stochastic rate of division in cells with mode $m=2$ depends on *Division Factor* concentration. In cell division, the parent cell size is halved between the two daughter cells and cells in the array are renumbered. The regulatory effect of *Division Factor* on cell division is described by Hill's function so that the probability of

cell division is minimal when *Division Factor* concentration is very low or very high and it is maximal at intermediate values of *Division Factor* concentration:

$$\left\{Cell(x_i, r_i, m_i = 2, a_i, DivF_i)\right\} \rightarrow \left\{ \begin{array}{l} Cell(x_l, \frac{r_l}{2}, m_l = 1, a_l, DivF_l),\ Cell(x_{l+1}, \frac{r_{l+1}}{2}, m_{l+1} = 1, a_{l+1}, DivF_{l+1}) \\ s_{l,l+1} = spring(c_l, c_{l+1})\} \rightarrow \{c_l, c_{l+1}, s_{l,l+1}\} \end{array} \right\}$$

$$\text{with } \rho_{div}(DivF_i) = \left(\frac{DivF_i}{k_{div,1}}\right)^{h_{div,1}} \Bigg/ \left(1 + \left(\frac{DivF_i}{k_{div,2}}\right)^{h_{div,2}}\right)$$

Where $k_{div,1}$ is the threshold of $DivF$ -dependent activation of cell division; $k^1_{d,y}$ is the threshold of $DivF$ -dependent inhibition of *Division Factor* synthesis; $h_{div,1}$ and $h_{div,2}$ are Hill coefficients which determine the response rate of these processes to the changes in *Division Factor* concentration. In addition, a renumbering of the cells is required (see the supplementary model).

d. Cell death

Cells on the tip of the root cap slough off during root growth. For simulation of this process, we consider in the model the process of cell death, but only for the first cell. The probability of “death” is coordinated with an average duration of cell cycle:

$$Cell(x_1, r_1, m_1, a_1, DivF_1) \rightarrow \{\}$$
$$\text{with } \rho_{death} = \frac{1}{3 * CellCycleTime}$$

Text S3. The model parameters.

An estimation of the model parameters has been performed using the published experimental data. The auxin flow from the shoot per conventional time unit (*α=1 cu/tu*) was selected as a half of an average auxin concentration in a root cell. The other parameters were normalized with respect to this value. In the model, auxin and PIN1 concentrations are evaluated in concentration unit (*cu*). The degradation coefficient (K_d) was estimated as a 0.5% of an average auxin concentration in a cell per conventional time unit (*tu*). The diffusion parameter *D* was averaged as 7% of auxin concentration in a cell per *tu*.

The degree of nonlinearity of the mechanism underlying PIN1 activation (*h1*) was selected based on the observation that PIN1 is upregulated through Aux/IAA-ARF-dependent feedback [7-8]. It has been shown that ARF transcriptional factors mediate the primary response to auxin as dimers [9]. Consequently, we selected the Hill's coefficient $h_1=2$. Auxin also regulates the proteasome-dependent PIN degradation, but the exact mechanism of this process is unknown [10]. However, the efficiency of PIN1 degradation regulated by auxin is not very high, since the effect of PIN1 proteins decrease becomes evident only after more than a 100-fold increase in the auxin level [7]. For this reason, we selected an intermediate value of Hill's coefficient h_2=4.

Expression of DR5 transcriptional fusions has been shown to be proportionally responsive to a range of auxin concentrations [11]. Thus, for adjusting the values of model parameters as well as for verification of model calculations, we translated the qualitative data on auxin response to quantitative data on relative auxin concentrations in root cells [Additional files 3; 4: II]. The values of other parameters were determined by the coordinatewise descent method [12-14] so that the model solution (*N=50)* matches the auxin distribution pattern shown by Sabatini et al., (1999) [Additional file 4: II] [11].

The coordinatewise descent method

The robust set of parameters [Additional file 4: IV] was obtained by coordinatewise descent method with the purpose to find a set of parameters with high h_2. The method is implemented in the STEP+ software package [13-14]. Below is a short description of the main idea:

The model solution with the basic set of parameters that reproduces the auxin distribution pattern from the published experimental data [11] (Fig. 4c) were compared with the model solutions generated by varying one parameter while holding all others fixed.

For comparison of the model solution with basic set of parameters and generated by parameter variation solutions, we used the function *S(p)* of the distance between them

$$S(p) = \sqrt{\sum_{i=1}^{n} (x_i(p) - x_i^*)^2}$$

where x_i* – the auxin concentration in the cell *i* for model solution with basic set; and x_i(p) – the auxin concentration calculated in the model with a new set of parameters. Varying only one parameter p_i we found its value at which the *S(p)* is the minimal. When

the minimum of the function was reached, the same calculations were iterated for each of other parameters. After some iteration, we received the “robust set” of parameters [Additional file 4: IV].

The additional parameters estimation for the 1D extended model.

For the *1D extended model*, an additional set of parameters to describe description of cell dynamics was estimated [Additional file 4: III]. The main condition was to fit the experimental data on mitotic activity along the root central axis. First, *DivF* -regulated rates of cell division $p_{div}(DivF_i)$ was calculated in the *1D minimal model* and shown to qualitatively match to the experimental data [Additional file 5a]. Second, to investigate the profile of mitotic activity along the *in silico* growing root, we quantified the number of divisions for each cell number in the *1D extended model* solutions. Each simulation begins with 20 cells and terminates when the array reaches 100 cells in length. Data from 50 simulations was summarized in a histogram which shows a good agreement with the experimental data [Additional file 5b].

Text S4. The analysis of the 1D *minimal model* with different sets of parameters

We performed the whole model analysis for the two model variants: the 1D *minimal* and the *1D extended models*. The *1D extended model* allows us to study the dynamics of auxin distribution in growing root whereas the 1D *minimal model* is well-behaved for analysis of the model sensitivity to changes in the initial data or for varying the parameters.

Two model parameter sets [Additional file 4: III, IV] which give 1D *minimal model* solutions that agree well with experimental data on auxin distribution in root [11] were used for the model analyses. In all simulations (except those separately mentioned) the number of cells was N=50.

1. **The basic set of parameters [Additional file 4: II].**

The 1D minimal model sensitivity to varying the parameters

In the simulations we varied one parameter while holding all others fixed. Decrease in α, D, q_1 values or increase in K_0, q_2, q_3, K_d parameter values results in shift of the auxin concentration maximum towards the first cell following by its disappearance [Additional file 7]. The distal maximum is still observed when α>0.45, D>0.015, q_1>0.65 or K_d<0.007, K_0<0.65, q_3<5.1, q_2<120. By contrast, gradual increase in α, D, q_1, Kd values or decrease in K_0, q_2, q_3 values results in shift of the auxin maximum towards the root base. However, when α>1.2, q_1>1.3, K_0<0.13, q_3<2.9, q_2<5 or K_d<0.003, the model lost its stationary solution. Instead we observed fluctuations of auxin concentration in the middle of the root (cells 7-20) with several nonstable maxima (dashed lines on [Additional file 7: a-e]. Oscillations of auxin concentration in the individual cells are shown in Fig. S3j. Subsequent gradual changes of parameters α>1.6, q_3<2.3 or q_2<3 result in establishment of additional auxin maxima at the root base (in the cell N) altogether with oscillations in the middle of the root. When we took q_1>1.9, K_0<0.05 a steady state solution with two maxima, at the root tip and at the root end (blue lines on [Additional file 7]), again appeared. Increase in the value of D doesn’t yield any temporal fluctuations: we only observed spatial smearing the zone of high auxin concentration [Additional file 7c].

The 1D minimal model sensitivity to changes in initial data

By changing auxin concentration in the initial data, we simulated root treatment by exogenous auxin. First, we calculated a stationary distribution for *the minimal model* with the basic set of parameters starting from zero initial data ($a_i = 0$, $i = 1\ldots50$). In this way we obtained a stationary distribution ($a_i,\ i = 1,...,N$) with the auxin concentration maximum in the fifth cell $a_5 = \max_{i=1...N}(a_i)$, named the “0-distribution”. Starting from the 0-distribution, we elevated the auxin concentration in an individual cell of the array $a_k^{'} = a_k + \delta$. The resulting distribution was taken as the initial condition for calculating a new stationary distribution, named the “(k, δ)-distribution”. This experiment was repeated for $k = 1\ldots N$, δ = [1, 1000]. Analysis of the calculations has demonstrated that if δ < 4, then the (k, δ)-distribution coincided with 0-distribution independently of the

number *k*. At $\delta \geq 4$, the position of the auxin maximum shifted to the middle part of the root, $a_l = \max\limits_{i=1...N}(a_i), 6 \leq l \leq 11$.

In this way we found that the minimal model with the basic set of parameters has several stationary solutions which can be realized using different initial data. To estimate the number of possible stationary solutions, we used the method of continuation with respect to parameters in the STEP+ package [12-14] which allows calculating evolution of auxin concentration in the ith cell in response to gradual changing of the parameter value. The method allows determining the total number of stationary solutions (stable and unstable), with the same set of parameters. On [Additional file 7e] it is shown the result of application of the method of continuation with respect to parameter α for calculation the evolution in a_1 , a_3 , a_7 and a_{11}. 14 solutions: 7 stable and 7 unstable - were found as the number of crossings of the graphic with a vertical line at α = 1. All solutions are similar and have the auxin maximum at the root tip, but the maximum positions vary [Additional file 7k].

The 1D extended model behavior

When simulating the root growth from the three initial cells, in short roots the auxin concentration maximum is localized to the second cell. With the increase in root length, the distance from the first cell to the maximum increases to four cells and then remains constant [Additional file 6]. We assumed that highly stable auxin maximum in the model with basic set of parameters can define one-cell length QC, like in *A. thaliana* [11].

2. Robust set of parameters [Additional file 4: IV]

With the robust set of parameters, the 1*D minimal model* similarly reproduces the generation and maintenance of auxin distribution both in normal development and after root cutting. However we observed additional features in the model behavior, by comparison with simulation of other conditions [Additional file 10].

1D Minimal model sensitivity to varying the parameters

This analysis was partially described in our previous paper [15]. It was noticed that unlike the 1D *minimal model* with the basic set of parameters, the 1D *minimal model* with the robust set of parameters doesn't yield oscillatory solutions upon varying only one of the parameters (except *D* value). Instead, as a result of gradual changes in parameters we observed stationary solutions with additional maxima in the middle of the root or at the root base (see [Additional files 8, 10]) at $\alpha>1.1$, $q_1>1.3$, $K_0<0.23$, $q_3<2.9$, $q_2<7$, $K_d<0.0037$ and $\alpha>1.18$, $q_1>1.2$, $K_0<0.13$, $q_3<2.7$, $q_2<4$, $K_d<0.0032$, respectively

Unlike the model with basic set of parameters, we observed fluctuations in auxin concentration when the value of *D* was gradually increased (>0.12). Increase in *D* value may be considered as simulation of appending the additional unpolarized auxin transportation to the system.

The minimal model sensitivity to changes in initial data

We observed a substantial sensitivity of the *1D minimal model* to changes in initial data. For example, in the model with *N*=50 we found more than 1000 stationary solutions [15] and most of them had several maxima along the root in addition to the distal one:

$$a_k = \max_{i=1\ldots l-1}(a_i),\ a_l = \max_{i=k+1\ldots m-1}(a_i),\ a_m = \max_{i=l+1\ldots r-1}(a_i)\ldots,\ where\ 1 < k < l \le m \le r\ldots \le N$$

[Additional file 10]. The same sensitivity to initial data was observed in the *1D extended model*.

1D extended model behavior

Investigation of the 1D *extended model* behavior with robust set of parameters revealed differences in "anatomy" of the growing root compared to the model with the basic set of parameters.

First, the *1D extended model* requires more than seven cells in the initial data (comparing three cells for the 1D extended model with the basic set of parameters) to keep the distal auxin maxima at the distance from the root end from the very beginning of simulation to up to several hundred cells. If we define less than seven cells, the auxin distal maximum disappears but then is generated again [Additional file 10]. So the model with the robust set of parameters describes auxin distribution in plants with larger embryonic RAM, that contains more than 3 layers along the central axis.

Second, the position of the distal auxin maximum floats in simulation for $a_k = \max_{i=1...N}(a_i),\ where\, 10 \le k \le 25$. This behavior could simulate auxin distribution in cereal plants with thick roots and a bigger QC [Additional file 10].

References

1. Mjolsness E, Yosiphon G: **Stochastic Process Semantics for Dynamical Grammars.** Ann. Math. Artif. Intell 2006. **47**:329-395.
2. Yosiphon.G. **Stochastic Parameterized Grammars: Formalization, Inference and Modeling Applications.** PhD thesis, University of California Irvine, May 2009
3. **Yosiphon G. PhD thesis** [http://computableplant.ics.uci.edu/~guy/downloads/papers/thesis.pdf]
4. Tikhonov AN: **Systems of differential equations containing small parameters in the derivative.** Mat. Sbornik N S 1952. **31**:575–586.
5. Jonsson, H., Heisler, MG., Shapiro, BE., Meyerowitz, EM., and Mjolsness, E: **An auxin-driven polarized transport model for phyllotaxis.** Proc Natl Acad Sci USA 2006, **103**(5):1633-1638.
6. Eric Mjolsness. **The Growth and Development of Some Recent Plant Models: A Viewpoint.** *Journal of Plant Growth Regulation.* 2006, **25**: 270-277.
7. Vieten A, Vanneste S, Wisniewska J, Benkova E, Benjamins R, Beeckman T, Luschnig C, Friml J: **Functional redundancy of PIN proteins is accompanied by auxindependent cross-regulation of PIN expression.** Development 2005, **132**(20):4521-4531.
8. Sauer M, Balla J, Luschnig C, Wisniewska J, Reinöhl V, Friml J, Benková E. **Canalization of auxin flow by Aux/IAA-ARF-dependent feedback regulation of PIN polarity.** Genes & Dev 2006, **20**:2902-2911.
9. Ulmasov T, Murfett J, Hagen G, Guilfoyle TJ. **Aux/IAA proteins repress expression of reporter genes containing natural and highly active synthetic auxin response elements.** Plant Cell 1997. **9**(11):1963-1971.
10. Sieberer T, Seifert GJ, Hauser MT, Grisafi P, Fink GR, Luschnig C. **Post-transcriptional control of the Arabidopsis auxin efflux carrier EIR1 requires AXR1.** Curr Biol 2000. **10**(24):1595-1598.

11. Sabatini S, Beis D, Wolkenfelt H, Murfett J, Guilfoyle T, Malamy J, Benfey P, Leyser O, Bechtold N, Weisbeek P, Scheres B: **An Auxin-Dependent Distal Organizer of Pattern and Polarity in the *Arabidopsis* Root.** Cell 1999, **99**(5):463–472.
12. Fadeev SI, Likhoshvai VA, Kogai VV, Omelyanchuk NA: **About mathematical modeling of pattern of auxin distribution in plant roots.** Siberian Electronic Mathematical Reports 2008. **5**:25-41.
13. Fadeev S.I., Korolev V.K., Gainova I.A., Medvedev A.E. **The package STEP+ for numerical study of autonomous systems arising when modeling dynamics of genetic-molecular systems.** *In proceedings of the fifth Intern. Conf. on Bioinformatics of Genome Regulation and Structure (BGRS'2006): 22–28 June 2008; Novosibirsk, Russia*. Edited by N.A. Kolchanov and Ralf Hofestadt. Novosibirsk; 2006 (3):118-120.
14. **STEP+ software description [**http://www.bionet.nsc.ru/meeting/bgrs_proceedings/papers/2006/BGRS_2006_V2_021.pdf]
15. Likhoshvai VA, Omel'ianchuk NA, Mironova VV, Fadeev SI, Mjolsness ED, Kolchanov NA: **Mathematical model of auxin distribution in the plant root.** Russian journal of developmental biology 2007. **38**:446-456.
