# Supplementary figures and images for: A plausible mechanism for auxin patterning along the developing root
Source: BMC Syst Biol. 2010 Jul 21;4:98. doi: 10.1186/1752-0509-4-98 (PMC2921385; doi:10.1186/1752-0509-4-98)

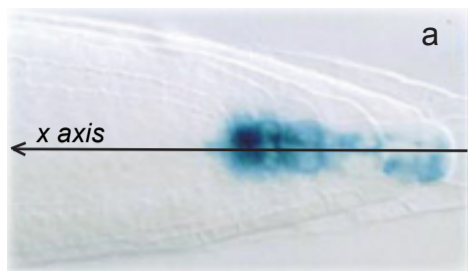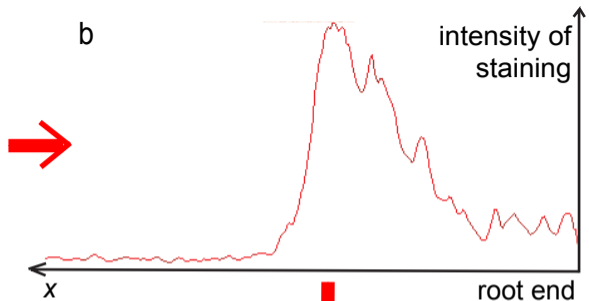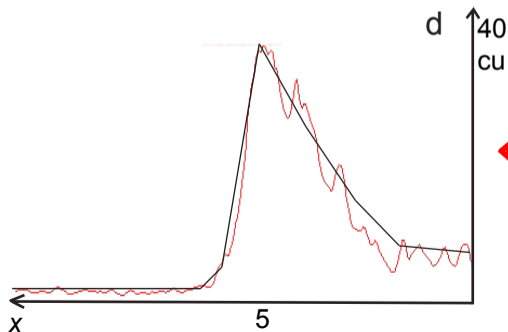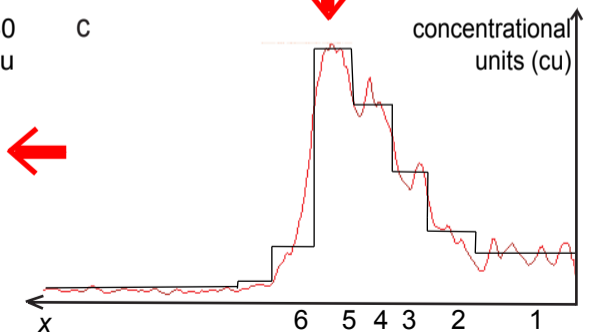

Supplement: Additional file 3 — Processing of the experimental data on auxin distribution in root. The figure showing the applied method of the experimental data on auxin distribution from DR5 auxin response images conversion to relative auxin concentrations in root cells. In the figure, (a) auxin distribution in the root according to DR5 reporter activity from Sabatini et al. (1999) [3]; (b) The result of processing image (a) using the ImageJ program, the intensity of staining along an image slice corresponding to the central root axis; (c) presenting this plot with cell layout and (d) model solutions matching the auxin distribution from (a), (b) and (c). [file 1752-0509-4-98-S3.PDF]

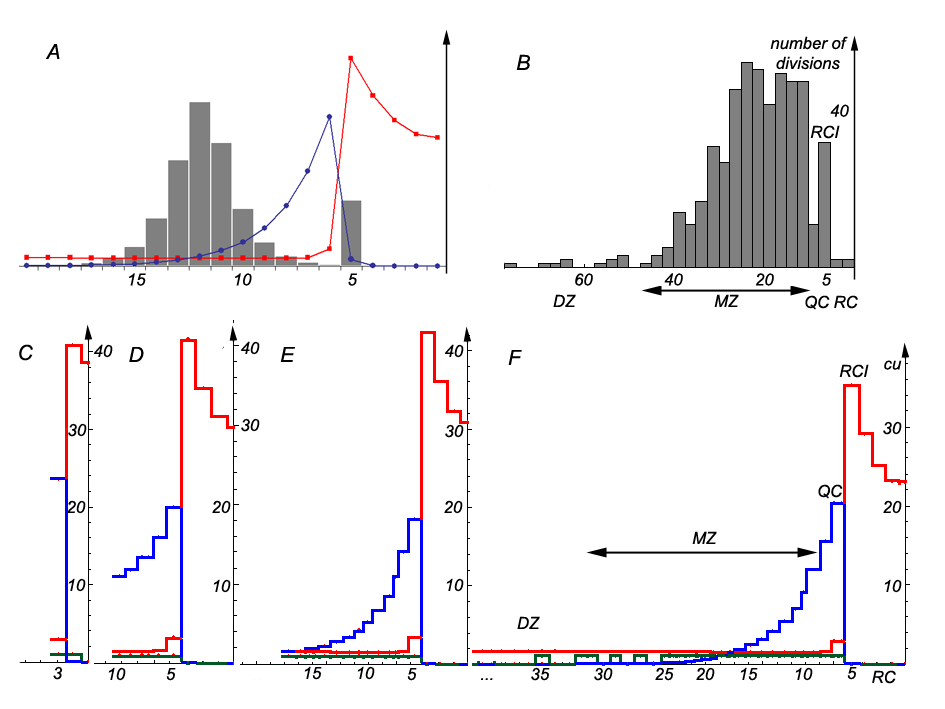

Supplement: Additional file 5 — Simulation of root growth along the central root axis. In the figure, (a) Distribution of auxin (red), Y (blue) and rates of cell division (gray columns) in conventional units along the central root axis. The curves were calculated in the 1 D minimal model with basic set of parameters. (b-d) The extended model solutions: (b) The mitotic activity along the central root axis; (c-f). Auxin and substance Y distribution. The green curve indicates the growth mode of cells (1- idle, 0- growth). c. the model was started from three cells; d. 10 cells; e. 20 cells; (f.) more than 100 cells. Cells of different types can be distinguished by considering both auxin concentration in the cell and its mitotic activity (see the main text for more details): QC - quiescent center; RCI - root cap initial; RC- root cap; MZ- meristematic zone; DZ- differentiation zone. [file 1752-0509-4-98-S5.TIFF]

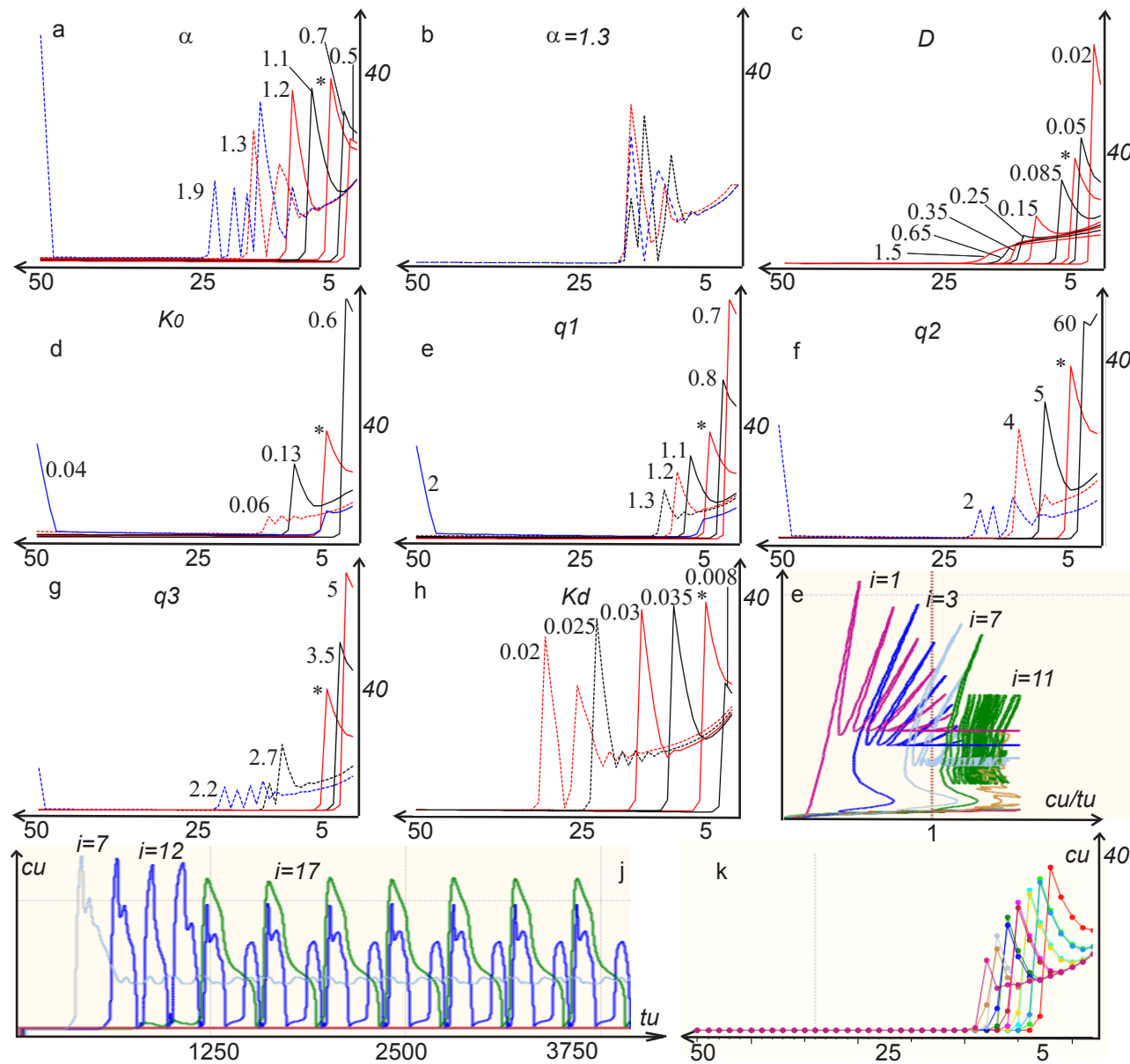

Supplement: Additional file 7 — Analysis of the 1D minimal model with basic set of parameters. The figure showing changes in the auxin distribution pattern at N = 50 in response to variations in: (a-b). α value; (c). diffusion rate (D) value; (d). K0 value; (e). q1 value; (f). q2 value; (g). q3 value; (h). Kd value, where other parameters were defined as in [Additional file 2: II]. Auxin distribution pattern calculated with the basic set of parameters and matching experimental data is marked by asterisk. Unstable fluctuations of auxin concentration in time are marked by dashed lines. The distributions having additional auxin maxima at the root base are blue colored. (e-k) The 1 D minimal model analysis in the STEP+ package [37]. e. The stationary solutions for ith cells obtained by the method of continuation with respect to parameter α. The number of crossings of the selected component with a vertical line at α = 1 corresponds to the total number of stationary solutions (stable and unstable), with the same set of parameters. k. The stationary solutions of the model estimated on figure (e.). j. Oscillation of auxin concentration in the ith cells in time (tu). In all plots, the y axis specifies auxin concentration in concentration units (cu). [file 1752-0509-4-98-S7.PDF]

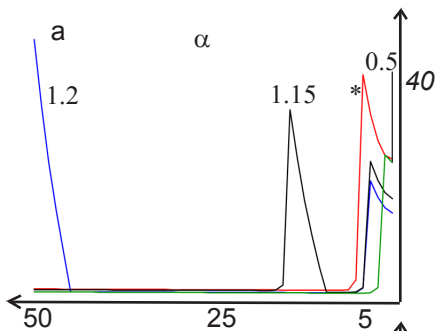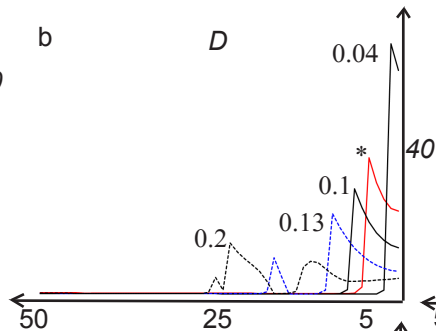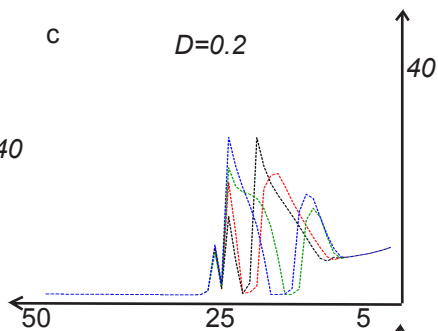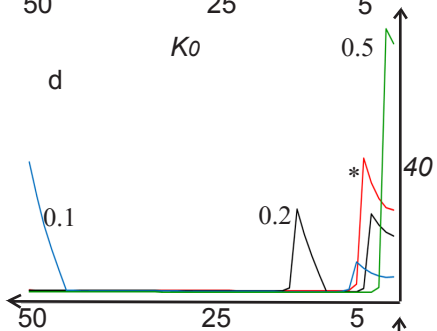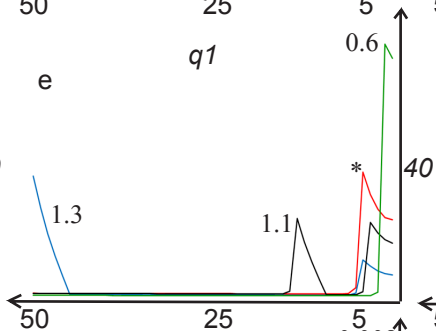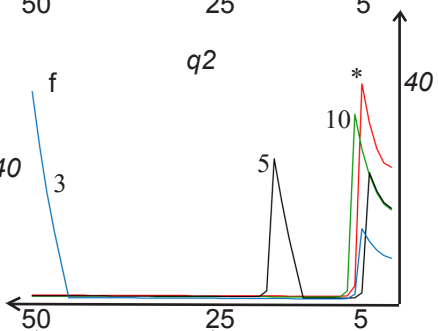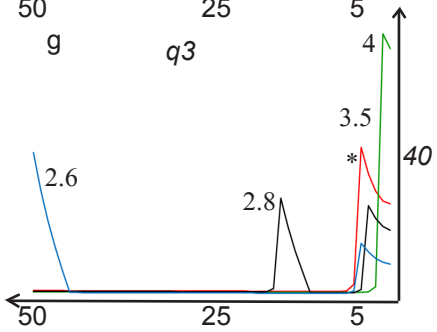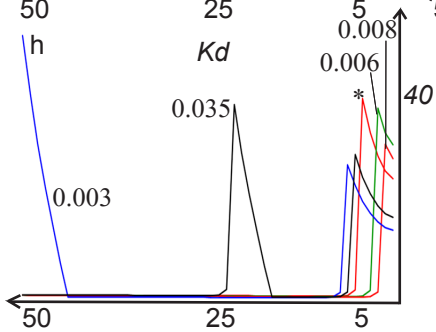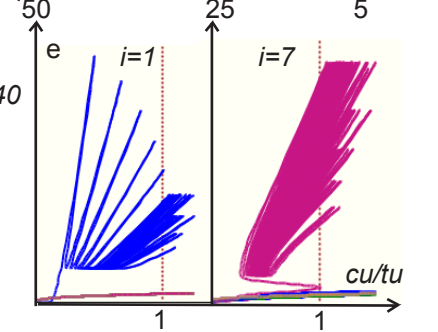

Supplement: Additional file 8 — Analysis of the 1D minimal model with robust set of parameters. The figure showing changes in the auxin distribution pattern at N = 50 in response to variations in: a. α value; b.-c. diffusion rate (D) value; d. K0 value; e. q1 value; f. q2 value; g. q3 value; h. Kd value, where other parameters were defined as in [Additional file 2: IV]. Auxin distribution pattern calculated with the robust set of parameter values and matching experimental data is marked by asterisk. Unstable fluctuations of auxin concentration in time are marked by dashed lines. The distributions having additional auxin maxima at the root base are blue colored. e. The 1 D minimal model analysis with the robust set of parameters in the STEP+ package [37]. The stationary solutions for ith cells are obtained by the method of continuation with respect to parameter α. The number of crossings (102) of the selected component with a vertical line at α = 1 corresponds to the total number of stationary solutions (stable and unstable), with the same set of parameters. In all plots, the y axis specifies auxin concentration in concentration units (cu). [file 1752-0509-4-98-S8.PDF]
