# Supplementary material for: A plausible mechanism for auxin patterning along the developing root
Source: BMC Syst Biol. 2010 Jul 21;4:98. doi: 10.1186/1752-0509-4-98 (PMC2921385; doi:10.1186/1752-0509-4-98)
Supplement: Additional file 4 — The model parameters. The table containing all model parameters. [file 1752-0509-4-98-S4.PDF]

Additional file 4. The parameters sets used in *the minimal and full models*.

I. The relative auxin concentrations in the cell array along the central root axis										
The cell number	10-50	9	8	7	6	5	4	3	2	1
The relative auxin concentration per cell (*cu*)	1.94	2	2	3.68	12.16	40	31.3	18	9	12.4

II. The basic set of parameters										
Parameter	α	D	K_d	K_o	q_1	q_2	q_3	h_1	h_2	$k1=k2$
Dimension	*cu/tu*	*1/tu*	*1/tu*	*1/tu*	*cu*	*cu*	*cu*	*dl*	*dl*	*1/tu*
Values	1	0.066	0.0043	0.193	1	6.73	3.26	2.13	4	1

III. The additional set of parameters for the full model											
Parameter	β	D_y	$k_{d,y}^0$	$k_{d,y}^1$	$k_{d,y}^2$	$h_{y,1}$	$h_{y,2}$	$k_{div.1}$	$k_{div.2}$	$h_{div,1}$	$h_{div,2}$
Dimension	*cu/tu*	*cu/tu*	*1/tu*	*cu*	*cu*	*dl*	*dl*	*1/tu*	*dl*	*dl*	*dl*
Values	1.5	0.1	0.3	3.5	0.5	4	1	0.1	0.01	2	3

IV. The robust set of parameters										
Parameter	α	D	K_d	K_o	q_1	q_2	q_3	h_1	h_2	$k1=k2$
Dimension	*cu/tu*	*1/tu*	*1/tu*	*1/tu*	*cu*	*cu*	*cu*	*dl*	*dl*	*1/tu*
Value	1	0.08	0.0045	0.25	1	100	3	2	10	1
