# Supplementary material for: A plausible mechanism for auxin patterning along the developing root
Source: BMC Syst Biol. 2010 Jul 21;4:98. doi: 10.1186/1752-0509-4-98 (PMC2921385; doi:10.1186/1752-0509-4-98)
Supplement: Additional file 10 — Comparison of the models behavior with basic and robust sets of parameters. The table showing the differences in the minimal and extended models behavior with basic and robust sets of parameters. [file 1752-0509-4-98-S10.pdf]

Additional file 10. Comparison of the model behavior with the basic and the robust sets of parameters.

Numerical experiments	***The basic set of parameters***	***The robust set of parameters***
**Estimation of the negative feedback from auxin to PIN1 expression**	10-fold decrease in PIN1 concentration observed when a >10 *cu*	10-fold decrease in PIN1 concentration observed when a >4.1 *cu*
**Simulation of root growth in the *full model***	The minimal number of cells in the initial data =3<br>Position of the distal maximum in root growth is stable:<br>$a_5 = \max_{i=1...N}(a_i)$ when $N > 15$	The minimal number of cells in the initial data =7<br>Position of the distal maximum in root growth is floating:<br>$a_k = \max_{i=1...N}(a_i)$, when $10 \le k \le 25$ and $N > 40$
**Conditions for appearance of oscillatory solutions in the *minimal model***	1) increase in auxin flow from the shoot α>1.2, or<br>2) decrease the rate of auxin active transport K_0<0.13, or<br>3) changes in coefficients of auxin regulated PIN1 expression q_1>1.3, q_3<2.9, q_2<5, or<br>4) decreased auxin dissipation rates K_d<0.003	1) increased auxin diffusion rates D>0.12
**Conditions for appearance of additional maximum in the inner part of the root**	The oscillatory solution found in the *minimal model* with increased value of auxin flow from the shoot (α) is transformed in the *full model* into periodical formation of additional auxin maxima.	1) increase in auxin flow from the shoot 1.1<α<1.18, or<br>2) decrease the rate of auxin active transport 0.013<K_0<0.23, or<br>3) changes in coefficients of auxin regulated PIN1 expression 1.2<q_1<1.3, 2.7<q_3<2.9, 4<q_2<7, or<br>4) decreased auxin dissipation rates 0.0032<K_d<0.0037, or<br>5) as a reply to changes in the initial data
**Conditions for appearance of additional maximum at the root base**	1) increase in auxin flow from the shoot α>1.6, or<br>2) decrease the rate of auxin active transport K_0<0.05, or<br>3) changes in coefficients of auxin regulated PIN1 expression q_1>1.9, q_3<2.3, q_2<3	1) increase in auxin flow from the shoot α>1.18, or<br>2) decrease the rate of auxin active transport K_0<0.13, or<br>3) changes in coefficients of auxin regulated PIN1 expression q_1>1.2, q_3<2.7, q_2<4, or<br>4) decreased auxin dissipation rates K_d<0.0032, or<br>5) as a reply to changes in the initial data
**The characteristics of the auxin transport system**	Distal auxin maximum is stable in root growth<br>Periodical formation of additional auxin maximum in the inner part of the root<br><br>Rare appearance of additional auxin maximum at the root base	Position of the distal auxin maximum is floating in root growth<br>Appearance of additional auxin maxima in the inner part of the root as a response to small changes in parameters or initial data<br>Frequent appearance of additional auxin maximum at the root base
**The root architecture under regulation of the auxin transport system**	**Taproot-like**	**Fibrous-like**
